# Innovation Competence in Healthcare: Individual, Environmental and Organisational Factors—A Mixed‐Method Systematic Review

**DOI:** 10.1111/jan.70396

**Published:** 2026-01-16

**Authors:** Mari Saukkoriipi, Outi Kanste, Erika Jarva, Pauliina Hyrkäs, Kristina Mikkonen

**Affiliations:** ^1^ Research Unit of Health Sciences and Technology University of Oulu Oulu Finland; ^2^ Medical Research Center Oulu Oulu University Hospital and University of Oulu Oulu Finland

**Keywords:** competence, healthcare, innovation, instruments, nursing, professional

## Abstract

**Aims:**

To identify healthcare professionals' experiences of innovation competence and the factors associated with it; and to examine the instruments developed to assess innovation competence and its associated factors among healthcare professionals.

**Design:**

A mixed‐methods systematic review.

**Methods:**

Researchers independently screened original studies by title and abstract (*n* = 2996) and then full text (*n* = 189). Eighteen studies were included: 16 quantitative and two qualitative. Qualitative data were analysed using inductive content analysis, and quantitative data were tabulated and synthesised narratively.

**Data Sources:**

The review followed the Joanna Briggs Institute Mixed Methods Systematic Review methodology. Searches were conducted in Scopus, CINAHL, Ovid Medline, ProQuest, Web of Science, PsycArticles, and Medic. Articles published in English or Finnish with no date restrictions were included. The search covered records from database inception to August 2024.

**Results:**

From qualitative studies, we identified three categories describing experiences of innovation competence: Competences for Innovation in Healthcare, Application and Impact of Innovation in Healthcare, and Challenges and Strategies for Implementing Innovation. Quantitative studies identified three conceptual domains: Individual Capacities in Innovation, Innovation‐related Competence Behaviours, and Social and Organisational Enablers. Four categories of factors associated with innovation competence emerged: sociodemographic, career‐related, organisational, and academic factors.

**Conclusions:**

Healthcare professionals' innovation competence is a multifaceted construct encompassing individual abilities, behavioural expressions, and social and organisational engagement. A systematic and multilevel approach that targets both personal attributes and organisational enablers is needed to strengthen competence. Enhancing innovation competence can improve the healthcare sector's ability to respond to complex challenges and sustain innovation capacity.

**Impact:**

Findings inform the development of education programmes and leadership strategies to enhance innovation competence among healthcare professionals, supporting innovation implementation in healthcare organisations.

**Patient or Public Contribution:**

No patient or public involvement was included in this study.

**Trial Registration:**

PROSPERO: CRD42024614551

## 1 Introduction

Healthcare worldwide faces major challenges that threaten the well‐being and health of populations at both the national and global levels. An ageing population, lack of traction in the healthcare sector, and low retention are examples of factors that create pressure on healthcare systems. Lack of traction refers to the declining attractiveness of healthcare professions and the difficulties in recruiting new professionals. Retention, in turn, highlights the challenges of maintaining a stable and committed workforce in the health sector. Rising costs further increase these pressures (Lucero‐Prisno et al. 2023; Ministry of Social Affairs and Health 2022). To address these challenges, innovations are needed to improve the quality of care, increase efficiency and productivity, and reduce costs (Gardner et al. 2010; Liu et al. 2020). These challenges and the need to strengthen innovation competence are relevant across health systems internationally.

The Oslo Manual defines innovation as a novel or enhanced product or process that represents a significant departure from the unit's previous products or processes. It becomes an innovation when it is introduced to potential users in the case of a product or implemented within the unit in the case of a process, and when it generates added value (OECD/Eurostat 2018). Healthcare innovations are new or improved solutions that aim to improve health outcomes, enhance quality of care and working conditions, and reduce costs, among other things (WHO 2023). However, innovation requires organisational and individual competencies to develop and implement new ideas effectively (OECD/Eurostat 2018).

Healthcare professionals play a key role in developing and implementing innovations, significantly influencing patient outcomes and organisational effectiveness (Bunpin et al. 2016; Yan et al. 2020). Nurses, recognised as the backbone of healthcare systems, deliver continuous patient care and are well positioned to drive innovation (Lahti et al. 2023). Their close interaction with patients, families, and communities provides a unique perspective on applying innovations to enhance health and manage conditions (Leary et al. 2022; White et al. 2016). However, to lead innovation effectively, they require specific training (Leary et al. 2022). In this study, healthcare professionals include nurses, midwives, public health nurses, physiotherapists, laboratory nurses, radiographers, dental hygienists, and occupational therapists, practitioners with practice‐oriented education and central roles in innovation implementation. Asurakkody and Shin (2018) define innovation as the creative implementation of new ideas, products, processes, and policies. Innovativeness entails openness to new ideas, experimentation, risk‐taking, and the ability to challenge existing processes (ANA 2017). It involves generating innovation by introducing diverse perspectives on a phenomenon and adapting to innovative thinking (Başkurt and Ateş 2020). Innovation competence builds upon these foundations and is understood as a multidimensional capability that integrates knowledge, skills, attitudes, and values, enabling active participation in innovation activities (Bozic Yams 2017; Hero et al. 2017). It encompasses creative thinking, social and leadership skills, project management, and the ability to identify and solve developmental challenges (Hero et al. 2017). Developing innovation competence can enhance healthcare quality, streamline service processes, and generate cost savings (Gardner et al. 2010; Liu et al. 2020).

A comprehensive definition of innovation competence includes future‐oriented thinking, creativity, social collaboration, and practical problem‐solving (Hero et al. 2017). It is a holistic capability that combines creativity, leadership, and commitment to transforming ideas into meaningful solutions (Tidd and Bessant 2009). Innovativeness is recognised as a social and communicative process that fosters the potential to generate novel and valuable ideas while developing ideas acquired from others (Bergendahl and Magnusson 2015). This study defines competence as the combination of knowledge, skills, attitudes, values, and behaviours necessary to successfully perform a task (Brophy and Kiely 2002; Rankin 2004). Competence is directly linked to work performance and can be systematically developed through education and standardised assessment (Bergevoet and Van Woerkum 2006).

Previous research in healthcare has identified key components of innovation competence, including the ability to develop and apply new methods in clinical work, adaptability to end‐user needs, and responsible innovation practices (BDO 2019; Pillay and Morris 2016; Tidd and Bessant 2009; Yan et al. 2018). Identified elements include knowledge acquisition, self‐development, practical innovation skills, attitudinal factors (e.g., resilience, self‐efficacy), social skills (e.g., teamwork), and behavioural competences such as opportunity recognition and design thinking (Dai et al. 2019; Pillay and Morris 2016). Innovation behaviour can be understood as a process encompassing idea discovery, generation, implementation, and advocacy (Bozkurt and Ercan 2022).

Despite growing recognition of the importance of innovation competence among healthcare professionals, research has struggled to distinguish between business skills and innovation skills, making it difficult to define and assess the necessary competences (White et al. 2016). Academic literature and training programs often emphasise business functions such as sales, production, finance, and performance measurement, which, while essential for organisational management, do not fully address the unique innovation needs of the healthcare sector (White et al. 2016).

Although the significance of innovation competence in healthcare has been acknowledged, research on the specific competences required in the innovation process remains limited. Previous studies have highlighted different levels of innovation capability (Dai et al. 2019) and professionals' willingness to share innovative ideas (Planas‐Campmany et al. 2020), yet a holistic understanding of innovation competence is lacking. There is also insufficient evidence regarding the specific components of innovation competence among healthcare professionals, as these factors have not been clearly defined based on empirical research.

Previous research has focused on the innovation competences of healthcare managers and organisations, with limited attention to frontline professionals (Birken et al. 2013; Hyrkäs et al. 2020; Pillay and Morris 2016; White et al. 2016). Yet, healthcare professionals are key drivers of change and can leverage innovation competence to improve services and outcomes (BDO 2019; National Academies of Sciences, Engineering, and Medicine 2021). Greater focus is needed on their role as part of organisational innovation, particularly for nurses, whose contributions are often overlooked (Leary et al. 2022).

No systematic review of instruments measuring innovation competence has been identified in the literature. Assessing innovation competence among healthcare professionals is essential for fostering innovation‐driven healthcare practices (Hero et al. 2021). Through instruments of innovation competence, factors influencing the innovative behaviour of healthcare professionals can be determined (Can et al. 2023). Understanding the characteristics and scope of these instruments is crucial for evaluating their effectiveness and identifying potential gaps in measurement. Therefore, gaining insights into their validity, reliability, and applicability in healthcare settings is essential.

While healthcare professionals are recognised as key drivers of innovation, existing research has largely focused on managerial and organisational levels, leaving frontline professionals' competences underexplored. Empirical definitions of innovation competence are lacking, and no prior systematic review has synthesised validated instruments for its assessment.

This review addresses these gaps by integrating healthcare professionals' experiences, identifying associated factors, and mapping available assessment instruments. The findings support competence‐based development, professional education, innovation‐oriented leadership, and guide future innovation policy. Prior to the review, relevant databases including PROSPERO were searched to confirm that no similar reviews were available or ongoing.

## 2 The Review

### 2.1 Aim

The aim of this systematic review was twofold. First, it sought to identify healthcare professionals' experiences of innovation competence and the factors associated with it. Second, it aimed to examine the instruments that have been developed to assess innovation competence and its associated factors among healthcare professionals.

The research questions were:

1. What are the experiences of healthcare professionals related to innovation competence?
2. What factors are associated with the innovation competence of healthcare professionals?
3. What types of instruments have been developed to assess the innovation competence and associated factors of healthcare professionals?

### 2.2 Methods

#### 2.2.1 Design

A mixed‐methods systematic review followed Joanna Briggs Institute guidelines (Aromataris and Munn 2020). The review included both original qualitative and quantitative studies. Its protocol has been registered in the PROSPERO database of the National Institute of Health Research (CRD42024614551). The results of this review were described based on the Preferred Reporting Items for Systematic Reviews and Meta‐analyses (PRISMA) checklist (Moher et al. 2009).

#### 2.2.2 Search Methods

A literature search was conducted in seven electronic databases (Scopus, CINAHL, Ovid Medline, ProQuest, Web of Science, PsycArticles and Medic) in the autumn of 2024. Search terms were defined according to PiCo and PEO inclusion and exclusion criteria before the database search was performed. A preliminary search was conducted to define the keywords for the article. This initial exploration helped identify relevant terms and refine the search strategy. At this stage of the research, a library information specialist was used to identify relevant search terms and strategy.

#### 2.2.3 Inclusion and Exclusion Criteria

The inclusion and exclusion criteria for qualitative and quantitative research were based on the PiCo (Participants, Phenomena of interest and Context; Lizarondo et al. 2020) and PEO (Participants, Exposure of Interest, Outcomes; Moola et al. 2020) guidelines (Table 1; File S1).

**TABLE 1:** Inclusion and exclusion criteria.

Criteria	Inclusion	Exclusion
*PEO framework*		
P (Participants)	Healthcare professionals (nurse, midwife, public health nurse, physiotherapist, laboratory nurse, x‐ray nurse, dental hygienist, occupational therapist)	Other healthcare professionals (doctor, dentist, pharmacist, psychologist, speech therapist, nutritionist, optician, dental technician, healthcare teacher, educator, healthcare student, healthcare leader, manager)
E (Exposure)	Factors associated with innovation competence and different types of measurements of innovation competence	Factors which are not associated with innovation competence and measurements focusing on other than healthcare professionals' innovation competence
O (Outcome)	Innovation competence areas	Outcomes related to innovative or creative approaches, innovative education methods, but not specifically to healthcare professionals' innovation competence
*PICo framework*		
P (Participants)	Healthcare professionals (as defined above)	Other healthcare professionals (as defined above)
i (Phenomenon of Interest)	The manifestation and development of innovation competence	Studies focusing on concepts other than healthcare professionals' innovation competence
Co (Context)	Healthcare settings (hospitals, primary healthcare, specialised healthcare, home care)	Studies not focused on healthcare, or studies primarily focusing on education
Type of studies	Qualitative, quantitative and mixed methods peer‐reviewed original studies	Reviews, editorials, expert opinion, case series, and studies that are not original peer‐reviewed studies

In qualitative and quantitative studies, eligible participants were healthcare professionals such as nurses, midwives, public health nurses, physiotherapists, laboratory nurses, radiographers, dental hygienists, and occupational therapists. To ensure relevance, only studies with at least 50% of participants representing these healthcare professionals were included. For example, a study in which less than half of the participants were nurses or allied healthcare professionals was excluded, whereas studies in which the majority of participants belonged to the target groups were eligible. Doctors, dentists, and other specialists were excluded because their educational pathways, professional roles, and competence frameworks differ substantially from those of the healthcare professionals included in this review. The selected groups typically have closer and more continuous patient contact in everyday practice, and innovation competence in these contexts is more directly linked to patient care processes. Including physicians and dentists would have resulted in a highly heterogeneous sample, limiting the comparability of findings.

Although the search strategy covered a range of healthcare professions, the vast majority of eligible studies focused on nurses. No eligible studies were found that specifically addressed other professional groups or the phenomenon of interest, exposure, outcome, or context did not match the inclusion criteria.

The phenomenon of interest was innovation competence, its manifestation, development, associated factors and assessment, within healthcare settings (hospitals, primary healthcare, specialised healthcare and home care). The included studies address healthcare professionals' innovation competence in areas such as initiating or implementing new methods, processes, or practices within healthcare settings, as well as developing the competences required to participate in or lead innovation activities.

The literature search did not set a time limit, but to be eligible, a study had to be peer‐reviewed and published in Finnish or English. No time restrictions were applied because research on innovation competence in healthcare is still emerging, and imposing time limits could have led to the exclusion of valuable early studies. The studies that met the inclusion criteria were all published between 2007 and 2023, which confirmed that the absence of time limits did not introduce bias. The choice of these two languages was based on the research team's language proficiency and to ensure that potentially relevant studies published nationally in Finland would not be overlooked, in addition to the global reach of English publications. The search terms were combined with Boolean operators (AND, OR, NOT).

#### 2.2.4 Search Outcome

**FIGURE 1:**
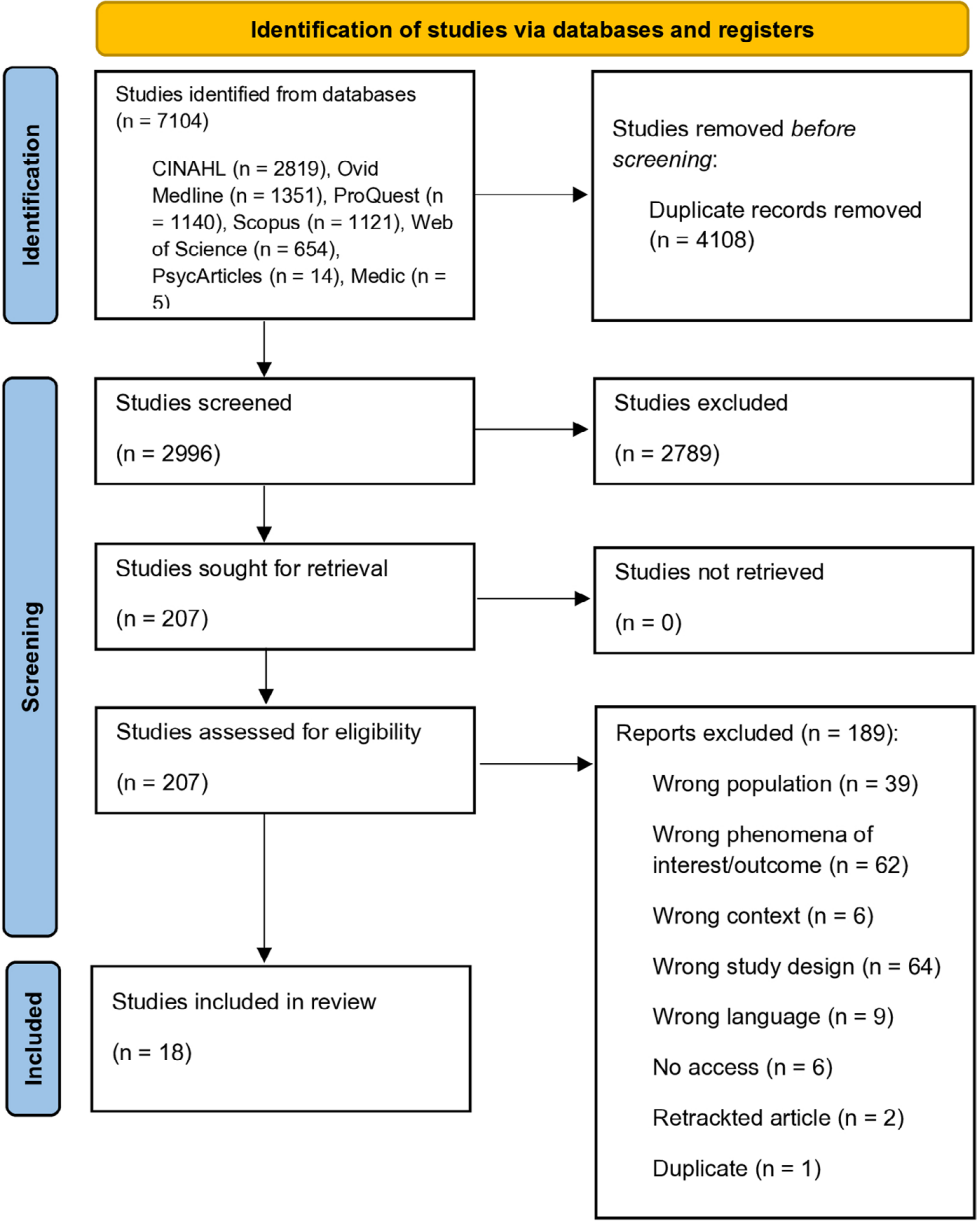
PRISMA flow diagram. Flow of studies through the identification, screening, eligibility, and inclusion phases of the systematic review.

The database search yielded 7104 publications (Figure 1). In total, 4108 studies were identified as duplicates, after which 2996 studies were screened by researchers separately (M.S., O.K., E.J., P.H., K.M.). Each researcher conducted this screening process independently, and after screening, the researchers compared and discussed their results until agreement was reached. 2996 studies were screened by title and abstract. Of these, 2789 were irrelevant. In the complete text review phase, 189 studies were screened, of which 18 were selected for final synthesis. Of these 18 studies, 16 were quantitative and two were qualitative.

#### 2.2.5 Quality Appraisal

The methodological quality of the 18 selected studies was evaluated according to JBI guidelines. The Critical Appraisal Checklist for Analytical Cross‐sectional Studies was used for the 16 quantitative studies (Moola et al. 2020), while the JBI Qualitative Assessment Research Instrument was used for two qualitative studies (Lockwood et al. 2020). The methodological quality of the studies was assessed by researchers (M.S., O.K., E.J., P.H., K.M.). In cases of disagreement during the assessment, the researchers discussed the issue until an agreement was reached. The results of the quality assessment are summarised in File S2. Among the quantitative studies, ten received the maximum score of 100% (Abdelwahab Ibrahim El‐Sayed et al. 2024; Afsar et al. 2018; Aoun et al. 2018; Kim and Park 2015; Lin et al. 2023; Li‐Ying et al. 2016; Song et al. 2023; Toscano et al. 2023; Tsai et al. 2013; Weng et al. 2012). Two studies scored 87.5% (Gomes et al. 2015; Mura et al. 2016), two scored 75% (Binnewies et al. 2007; Timmermans et al. 2013), one scored 62.5% (Wicaksono et al. 2023), and one scored 50% (Aoun and Hasnan 2017). Among the qualitative studies, one achieved a score of 100% (Wang et al. 2023), while another scored 70% (Oliveira et al. 2023).

#### 2.2.6 Data Abstraction

The following data were extracted from all of the studies included in the review: author(s), year of publication, country of origin, study purpose, participants and settings, study design, methods of data collection and analysis, instrument, phenomenon of interest/outcome, factors associated with outcomes and key findings (Table 2).

**TABLE 2:** Data extraction.

Author(s), year of publication, country	Study purpose	Participants and settings, study design	Methods of data collection and analysis, and the instruments	Phenomenon of interest/Outcome	Key findings
				Factors associated with outcomes	
Abdelwahab Ibrahim El‐Sayed et al. (2024), Egypt	To assess the effect of supervisor knowledge sharing behaviour and organisational absorptive capacity on nurses' creativity	Nurse (*n* = 700), hospital. A cross‐sectional study	Self‐administered printed questionnaires. Supervisor knowledge sharing behaviour scale, Organisational absorptive capacity questionnaire, Creativity self‐assessment questionnaire	Nurses' creativity The effect of supervisor knowledge sharing behaviour and organisational absorptive capacity	Supervisor knowledge sharing behaviour and absorptive capacity play an important role in stimulating nurses' creativity. Supervisor knowledge sharing behaviour and absorptive capacity are powerful antecedents for nurses' creativity
Afsar et al. (2018), Thailand	To investigate the impact of a nurse's person‐organisation (P‐O) fit on his/her innovative work behaviour (IWB) and understand the psychological mechanisms surrounding this relationship	Nurse (*n* = 441), public sector hospital. A cross‐sectional study	Questionnaire, Confirmatory factor analysis (CFA). Person‐organisation (P‐O) fit scale, empowerment at work scale, ten‐item scale measuring innovative work behaviour (IWB), and the eight‐item scale in the knowledge sharing behaviour (KSB)	Innovative work behaviour Nurse's person‐organisation (P‐O) fit, psychological empowerment, knowledge sharing behaviour (KSB)	The study indicates that nurses' person‐organisation (P‐O) fit is positively related to both self‐ratings of innovative behaviours and that psychological empowerment mediates this relationship. A nurse's perception of value congruence impacts his/her perception of feeling of empowerment, which in turn helps engage him/her in acts of innovativeness more often. The relationship between person‐organisation (P‐O) fit and innovative work behaviour (IWB) is stronger among nurses who frequently share their best practices and mistakes with co‐workers
Aoun and Hasnan (2017), Malaysia	To determine the influence of implementing soft total quality management (TQM) on the innovation skills (INS) of employees	Nurses (*n* = 380), public and private accredited hospitals. A cross‐sectional study	Questionnaire. Innovation skills (INS), Continuous improvement (CI), people‐based management (PBM)	Innovation skills (INS) of employees Total quality management (TQM)	Soft total quality management (TQM) significantly and positively influences innovation skills (INS). People‐based management (PBM) significantly and positively influences innovation skills (INS)
Aoun et al. (2018), Malaysia	To study the relationship between lean practices, soft total quality management (TQM) and innovation skills in Lebanese hospitals	Nurses (*n* = 352), private and public hospitals. A cross‐sectional study	Self‐administered questionnaire. Structural equation modelling (SEM). Study, don't tell	Innovation skills Lean practices, soft total quality management (TQM)	Lean practices significantly influenced the innovation skills; however, soft total quality management (TQM) did not mediate this relationship because it was not well implemented, especially at the level of people‐based management and continuous improvement
Binnewies et al. (2007), Germany	To provide a more detailed view of the creative process	Nursing staff (*n* = 52), health care. A cross‐sectional study	Questionnaire and interview. Personal initiative at work scale, Idea creativity, Idea‐related communication, Internal or external problem identification, Problem identification, Idea generation	Creative process —	Idea‐related communication was positively related to engagement in the creative process but not idea creativity. Personal initiative boosted engagement at the beginning of the creative process and was positively related to creativity. Results suggest that idea‐related communication is essential for showing engagement in the creative process. In contrast, personal initiative is vital at the beginning of the creative process and for idea creation
Gomes et al. (2015), Portugal	To explore the relationship between self‐leadership, work engagement and individual innovation	Nurses (*n* = 288), healthcare units (surgical, medical, mixed). A cross‐sectional study	Revised Self‐Leadership Questionnaire, Utrecht Work Engagement Scale, Individual Innovation scale	Individual innovation Self‐leadership, work engagement	A study shows a positive relationship between self‐leadership, work engagement and individual innovation. Furthermore, the results showed that work engagement had a mediating effect on the relationship between self‐leadership and individual innovation. Overall, this research will contribute towards gaining more insights into the role of self‐leadership and work engagement in individual innovation
Kim and Park (2015), South Korea	A study identified the factors that affect the innovative behaviours of nurses at general hospitals based on their individual and organisational characteristics	Registered nurses (*n* = 347), general hospitals. A cross‐sectional study	Self‐report questionnaire, Descriptive statistics. Individual innovative behaviours scale, Self‐leadership scale, Individual knowledge sharing scale, Creative self‐efficacy scale, Organisational knowledge sharing scale, Innovative organisational culture scale	The innovative behaviours of nurses —	Self‐leadership, creative self‐efficacy, and individual knowledge sharing directly affected individual innovative behaviours. Organisational knowledge sharing indirectly affected individual innovative behaviours, and this effect was mediated by an innovative organisational culture
Lin et al. (2023), China	To investigate the innovative behaviour and information literacy of emergency specialist nurses and analysed the influence of information literacy on innovative behaviour to promote the latter	Emergency specialist nurses (*n* = 484), hospitals (including secondary, lower and tertiary hospitals). A cross‐sectional study	Online questionnaire. Univariate analysis, Pearson's analysis, and multiple linear regression. Innovative behaviour scale for nurses, Information literacy questionnaire	Innovative behaviour Information literacy	Literacy positively correlated with innovative behaviour. Experience in publishing academic papers influenced innovative behaviour
Li‐Ying et al. (2016), Denmark	To investigate the influence of intensive care unit nurses' knowledge sharing behaviour on nurse innovation, given different conditions of care quality control	Nurses (*n* = 180), hospitals. A cross‐sectional study	Web‐based questionnaire. The employee version: four general innovation performance items, knowledge sharing behaviours developed. Manager version: Measures of control of care quality	Nurse innovation Knowledge sharing behaviour	Different aspects of knowledge sharing affect innovation differently, depending on the strength of the control of care quality within the unit. Knowledge sharing among ICU nurses is positively associated with individual nurse innovation. Furthermore, this positive relationship holds for all three types of knowledge sharing: written contributions, organisational communication and personal interaction
Mura et al. (2016), Italy	To provide arguments and empirical evidence that different knowledge sharing behaviours—i.e., sharing best practices, sharing mistakes, seeking feedback are promoted and enabled by different types of knowledge assets, and differently affect employees' innovative work behaviours	*N* = 129, nurses (*n* = 69), physiotherapists (*n* = 7), other healthcare operators (*n* = 53), hospice and palliative care organisations. A cross‐sectional study	Structural social capital scale, Organisational capital scale, Knowledge sharing block – sharing best practices scale, Innovative work behaviour (IWB) scale	Employees' innovative work behaviours Different knowledge sharing behaviours, different types of knowledge assets	The linkage between knowledge assets and knowledge sharing is both direct and indirect, with psychological safety as a relevant mediating construct. The linkage between relational and structural social capital and seeking feedback and sharing mistakes is fully mediated by psychological safety. Each dimension of knowledge sharing affects the different dimensions of employees' innovative work behaviour—i.e., idea generation, idea promotion, idea implementation—distinctly. While sharing of best practices influences all of them, seeking feedback affects idea promotion and sharing mistakes influences idea implementation
Oliveira et al. (2023), Brazil	To explore the managerial competencies engaged in innovative actions among Brazilian nurses working in primary health care	*N* = 76, clinical nurses (*n* = 46), managerial nurses (*n* = 30), primary health care. A qualitative, exploratory, descriptive approach	Semistructured and audiorecorded interviews. Data were processed in IraMuTeQ 0.7 and later analysed by descending hierarchical classification	Nurse managers' innovation competence —	Managerial competencies that influenced the development of innovative actions implemented were communication and teamwork in planning innovative actions, continuing education for application and implementation, and leadership and people management
Song et al. (2023), China	To analyse the correlations among organisational justice, knowledge‐hiding behaviour and nurses' innovation ability	*N* = 1486 Team leader (*n* = 121), research specialist staff (*n* = 15), key staff (*n* = 255), general nurse (*n* = 1094), hospitals. A cross‐sectional study	Questionnaire. Nursing innovation behaviour scale, knowledge‐hiding behaviour scales and organisational justice scales	Nurses' innovation ability Organisational justice, knowledge‐hiding behaviour	Team role, nursing age, number of training, literature‐reading habits, organisational justice, information justice, fair distribution and deaf knowledge hiding as the influencing factors of nurses' innovation. Nurses' sense of organisational fairness negatively correlated with knowledge concealment and positively correlated with innovation ability. Moreover, knowledge hiding is negatively correlated with nurses' innovation ability. Furthermore, knowledge‐hiding plays a partial intermediary role between organisational fairness and nurses' innovation ability
Timmermans et al. (2013), Belgium	To study the relationship between team learning activities and the implementation effectiveness of innovations in nursing teams	Nurses (*n* = 469), university hospital and mental health institutions. A cross‐sectional study	Team learning scale for nursing teams, intervention fidelity scale, the Lowry‐Jopp Neuman Model Evaluation Instrument (LJNMEI)	Implementation effectiveness of innovations Team learning activities	Correlation analyses revealed positive relationships between the team learning activities, handling production‐oriented information, and the implementation effectiveness of an incremental innovation. In addition, team learning activities about development‐oriented information positively affected the implementation of a radical innovation
Toscano et al. (2023), Italy	To advance the current understanding and the knowledge available about the relationship between creative style and innovation behaviours in the specific working population of nurses. To shed light on how emotion‐regulation strategies, such as positive reappraisal and putting into perspective, can facilitate or hinder turning novel and original ideas into actual and practical innovation, thus acting as underlying psychological mechanisms of innovation at work. To provide insights into managerial actions that might be deployed to enhance emotion regulation among nurses, with the broader objective of promoting innovation within healthcare organisations	Nurses (*n* = 187), hospital. A cross‐sectional study	Questionnaire. Problem‐solving style questionnaire (PSSQ), Short cognitive emotional regulation questionnaire (CERQ‐short), modified scale nurses' innovative behaviours	Innovation behaviours Creative style, emotion‐regulation strategies, managerial action	Results show that positive reappraisal completely mediates the relationship between creative style and innovative behaviours, while putting into perspective moderates the relationship between positive reappraisal and innovative behaviours
Tsai et al. (2013), Taiwan	To understand the relationship of individual characteristics, perceived worksite support and perceived personal creativity to clinical nurses' innovative outcome	*N* = 538, awarded nurses (*n* = 32), non‐awarded nurses (*n* = 506), Hospitals. A cross‐sectional study	A self‐administered questionnaire. Modified worksite support scale, creativity scale	Clinical nurses' innovative outcome Individual characteristics, perceived worksite support, and perceived personal creativity	The level of creativity perceived by all participants was moderate to high. Individual characteristics and worksite support were both correlated with perceived creativity. Individual characteristics and worksite support showed some correlation as well. After controlling for demographic variables, individual characteristics and worksite support could predict perceived creativity, but only individual characteristics affected the innovative outcome. Perceived creativity did not have mediation effects either between individual characteristics and innovative outcome or between worksite support and innovative outcome
Wang et al. (2023), China	To analyse MNS postgraduates' experiences in their training in innovative practical ability	*N* = 18, MNS students (*n* = 12), MNS nurses (*n* = 6), university and affiliated tertiary hospitals. A qualitative research	Semi‐structured face‐to‐face interviews. Analysed by Colaizzi's 7‐step method	Experiences in innovative practical ability —	Three key themes emerged: (1) the cognition of innovative practical ability; (2) the experience of cultivating innovative practical ability at school; and (3) the experience of cultivating innovative practical ability in the hospital
Weng et al. (2012), Taiwan	To explore the cross‐level effects of the four dimensions of patient safety climate on nursing innovation	Nurses (*n* = 808), hospital. A cross‐sectional study	Questionnaire. Patient safety climate scales, knowledge creation scale, innovation behaviour scale, innovation diffusion scale	Nursing innovation Four dimensions of patient safety climate	Of these three dimensions of nursing innovation, the nurses perceived the level of knowledge creation as the highest. Regarding patient safety climate, managerial practices regarding patient safety scored the highest, followed by patient safety procedures, patient safety information flow and patient safety priority. Only patient safety information flow significantly positively influenced knowledge creation, innovation behaviour or innovation diffusion
Wicaksono et al. (2023), Indonesia	To analyse the influence of Islamic work ethics on nurse innovation capability with knowledge sharing behaviour as intervening variables	Nurses (*n* = 154), a sharia hospital. A cross‐sectional study	Individual Innovation Capability Scale, Knowledge Sharing Behaviour Scale (KSB‐8), 8‐Item Islamic Work Ethics	Nurse innovation capability Islamic work ethics, knowledge sharing behaviour	Islamic Work Ethics had a positive and insignificant effect on the Innovative Ability of Nurses; Islamic Work Ethics have a positive and significant effect on Knowledge Sharing Behaviour; Knowledge Sharing Behaviour has a positive and significant effect on the Innovation Ability of Nurses

#### 2.2.7 Synthesis

An inductive content analysis was conducted on the two qualitative studies to summarise the findings describing healthcare professionals' experiences related to innovation competence (Mikkonen and Kääriäinen 2020). At the beginning of the analysis, the raw text was divided into units of meaning (single words, sentences or entities) based on the research question. A total of 34 codes related to the research question were generated from the meaning units. In the next step of the content analysis, three categories were identified out of nine sub‐categories (Table 3).

**TABLE 3:** Healthcare professionals' experiences related to innovation competence.

Code	Sub‐categories	Categories
Adapting innovative activities to work activities	Innovation competence in integrating innovation into work processes	Competences for Innovation in Healthcare
Team workflow is considered in innovative activity planning		
Team organisation is considered in innovative activity planning		
Communication is crucial in planning innovations	Innovation communication skills	
Teamwork is crucial in planning innovations	Innovation teamwork ability	
Team knowledge is key to service improvement through innovations		
Managerial skills are essential to implement innovations	Innovation management and leadership skills	
Management skills impact roles in innovation		
Leadership is fundamental for innovation		
People management is fundamental for innovation		
Need a strategic vision to organise teams to innovate		
Local leadership is a pillar of innovation		
People management is a pillar of innovation		
Problem identification is essential	Critical thinking and problem‐solving skills for innovation	
Critical thinking is important for innovation		
Mentors are important in developing innovative skills	Development of innovation competence	
Innovative thinking is trained in clinical training		
Cultivating innovative skills is crucial for postgraduate education		
Cultivating innovative skills is crucial for hospital research		
Innovative practical ability is important in a project application	Practical application of innovation in healthcare	Application and Impact of Innovation in Healthcare
Innovative practical ability is important in project implementation		
Innovative practical ability is important in quality control		
Innovative thinking is translated into practice to solve clinical problems		
Innovative skills are used to solve clinical problems		
Innovative skills improve nursing methods	Impact of innovation on healthcare practice	
Innovative skills improve nursing processes		
Innovative skills improve nursing efficiency		
Combining innovative skills with clinical practice is crucial		
Translating innovative thinking into practice is challenging	Challenges in implementing innovation	Challenges and Strategies to Implementing Innovation
Routine nursing work hinders the development of innovative thinking		
Identifying problems is easier than implementing innovative ideas	Strategies for overcoming innovation barriers	
Balancing innovative skill development with daily nursing work is important		
The use of innovative skills depends on the intentions of nursing supervisors		

The 16 quantitative studies were tabulated and synthesised to identify statistically significant factors associated with the innovation competence of healthcare professionals (Popay et al. 2006). This involved collecting all the narrative results from the selected studies, reducing the data by identifying similarities and dissimilarities, and organising similar findings into meaningful classifications (Table 4).

**TABLE 4:** Factors associated with the innovation competence of healthcare professionals.

Outcomes										
	Individual capacities in innovation				Innovation‐related competence behaviours				Social and organisational enablers in innovation competence growth	
Factors	Psychological empowerment	Information literacy	Individual characteristics	Self‐leader‐ship	Knowledge sharing behaviour	Knowledge sharing behaviour	Knowledge‐hiding behaviour	Work engagement	Organisational justice	Nurse's person‐organisation fit
Participants (*n*)	*n* = 441	*n* = 484	*n* = 538	*n* = 288	*n* = 441	*n* = 180	*n* = 1486	*n* = 288	*n* = 1486	*n* = 441
*Sociodemographic characteristic*										
Age	NS	NS	***p* < 0.001**	***p* < 0.01**	NS	***p* < 0.5**	***p* < 0.001**	***p* < 0.01**	***p* < 0.001**	NS
Gender	***p* < 0.05**	NS	NS	NS	***p* < 0.05**	***p* < 0.5**	NR	NS	NR	***p* < 0.05**
Relationship status	NR	NR	***p* < 0.001**	NR	NR	NR	***p* < 0.001**	NR	***p* < 0.001**	NR
Have children or not	NR	NR	***p* < 0.001**	NR	NR	NR	NR	NR	NR	NR
Education	NR	***p* < 0.001**	NS	NR	NR	NR	***p* < 0.001**	NR	***p* < 0.001**	NR
*Career characteristic*										
Working years	NR	***p* < 0.001**	***p* < 0.001**	NR	NR	NR	***p* < 0.001**	NR	***p* < 0.001**	NR
Job tenure	***p* < 0.05**	NR	NR	***p* < 0.01**	***p* < 0.05**	***p* < 0.5**	NR	***p* < 0.01**	NR	***p* < 0.05**
Professional title	NR	***p* < 0.001**	NR	***p* < 0.01**	NR	NR	***p* < 0.001**	***p* < 0.01**	***p* < 0.001**	NR
Papers' reading time	NR	NR	NR	NR	NR	NR	***p* < 0.001**	NR	***p* < 0.001**	NR
Training and learning (> 1 day 1 time)	NR	NR	NR	NR	NR	NR	***p* < 0.01**	NR	***p* < 0.01**	NR
*Organisational characteristic*										
Number of working hours per week	NR	NR	NR	NR	NR	***p* < 0.5**	NR	NR	NR	NR
Team role	NR	NR	NR	NR	NR	NR	***p* < 0.001**	NR	***p* < 0.001**	NR
Hospital type	NR	NR	***p* < 0.001**	NR	NR	NR	NR	NR	NR	NR
*Academic*										
Experience in publishing academic papers	NR	***p* < 0.5**	NR	NR	NR	NR	NR	NR	NR	NR

*Note:* Bold values indicate statistically significantly associated (*p* < 0.05 to *p* < 0.001) with outcomes representing innovation competence.

Abbreviations: NR, not reported; NS, non‐significant.

A separate summary table (Table 5) was created to identify what instruments have been developed to assess the innovation competence and associated factors of healthcare professionals. The table includes the study reference, the specific instrument utilised, the assessment method (e.g., Likert scale), the aim of the instrument, a brief description of its content, and information regarding its validity and reliability.

**TABLE 5:** Instruments developed to assess the innovation competence and associated factors of healthcare professionals.

Category	Study	Instrument	Aim of the instrument, assessment	Instrument content	Content validity, construct validity, reliability
Individual innovation competence	Abdelwahab Ibrahim El‐Sayed et al. (2024)	Creativity self‐assessment questionnaire (Smith 2010)	To assess nurses' creativity. Likert scale ranging from 1 = strongly disagree to 5 = strongly agree. Higher scores indicate a high level of creativity	28 items divided into 4 subscales: Generating ideas (7 items) Digging deeper into ideas (7 items) Exploring ideas (7 items) Listening to the inner voice (7 items)	Expert panel (*n* = 7); pilot (*n* = 70). KMO = 0.853; Item loadings = 0.522–0.756. *α* = 0.710–0.792; Item‐total corr = 0.536–0.827
	Afsar et al. (2018)	Innovative work behaviour (IWB) scale (De Jong and Den Hartog 2010)	To measure innovative work behaviour. A 5‐point Likert scale	10 items	Content validity and construct validity not reported. *α* = 0.84
	Aoun and Hasnan (2017)	Innovation skills (INS)	NM A 6‐point Likert scale that ranged from 1 = strongly disagree to 6 = strongly agree	15 items	Pre‐tested by academic experts. Construct validity and reliability not reported
	Aoun et al. (2018)	A self‐administered questionnaire	The relationship between lean practices, soft total quality management and innovation skills. A 6‐point Likert scale that ranged from 1 = strongly disagree to 6 = strongly agree	NM	Pre‐tested. KMO > 0.5; CFI ≥ 0.90; GFI ≥ 0.90; AGFI ≥ 0.90; RMSEA ≥ 0.08; TLI ≥ 0.90; Bartlett's test of sphericity *p* < 0.05. *α* = 0.748–0.909
	Binnewies et al. (2007)	Idea creativity (Zhou 1998; Zhou and Oldham 2001)	To evaluate the creativity of ideas. A 10‐point scale ranging from 1 = not creative at all to 10 = extremely creative. Creativity was evaluated by three expert nursing educators using predefined criteria, including a definition and examples. Assessments considered novelty, usefulness, and overall creativity	NM	Content validity and construct validity not reported. ICC = 0.93
	Kim and Park (2015)	Creative self‐efficacy scale (Carmeli and Schaubroeck 2007)	To examine creative self‐efficacy. A 6‐point Likert scale from 1 = strongly disagree to 6 = strongly agree. Higher scores indicate greater levels of creative self‐efficacy	8 items	Content validity and construct validity not reported. *α* = 0.94
		Individual innovative behaviours scale (Kleysen and Street 2001)	To examine individual innovative behaviours. A 6‐point Likert scale from 1 = never to 6 = always. Higher scores indicate greater levels of innovative behaviours	14 items divided into five subscales: Opportunity exploration Generativity Formative investigation Championing Application	Content validity and construct validity not reported. *α* = 0.95
	Lin et al. (2023)	Innovative behaviour scale for nurses (Bao et al. 2012)	To evaluate the innovative behaviour of nurses. A 5‐point Likert scale from 1 = never to 5 = very frequently. The higher the total score and average score of the scale and all dimensions, the higher the nurses' innovative behaviour	10 items divided into 3 subscales Generating ideas (3 items) Obtaining support (4 items) Realising ideas (3 items)	Expert consultation. Construct validity not reported. *α* = 0.746–0.907
	Mura et al. (2016)	Innovative work behaviour (IWB) scale (De Jong and Den Hartog 2010)	To indicate the extent to which individuals are creative and develop new ideas, promote them with and seek endorsement from co‐workers, and seek to implement them within their organisation's routines. A 7‐point Likert scale	12 items in 3 subscales: Idea generation Idea promotion Idea implementation	Pilot study. Construct validity not reported. CR = 0.802–0.881
	Song et al. (2023)	Nursing innovation behaviour scale (Bao et al. 2012) adapted to the Chinese setting	To measure nurses' innovation ability. A 5‐point Likert‐type scale from 1 = never to 5 = very frequently. The highest score showed the highest nursing innovation behaviour	10 items divided into 3 dimensions: Generating ideas Gaining support implementing ideas	CVI = 0.910. Construct validity not reported. *α* = 0.879–0.948
	Toscano et al. (2023)	Modified scale nurses' innovative behaviours (George and Zhou 2001)	To measure supervisors' assessment of nurses' innovative behaviour. A 5‐point Likert‐type scale from 1 = Not at all characteristic to 5 = very characteristic. A a higher score indicated a higher level of innovation behaviours. One supervisor rated each nurse	3 items.	Content validity not reported. AVE = 0.58. *α* = 0.80
	Tsai et al. (2013)	Employee creativity scale (Lin et al. 2007)	To measure perceived creativity. A 5‐point Likert‐type scale from 1 = strongly disagree to 5 = strongly agree	8 items	CVI = 1.00 (3 experts). Factor analysis supported subscales. *α* = 0.93
	Weng et al. (2012)	Knowledge creation, innovation behaviour and innovation diffusion scale (Smith et al. 2005; Hansen and Birkinshaw 2007; Scott and Bruce 1994; Rogers 1995)	To measure nursing innovation. A 5‐point Likert scale from 1 = do not agree very much to 5 = strongly agree	23 items divided into 3 subscales: Knowledge creation (9 items) Innovation behaviour (8 items) Patient safety priority (6 items)	Evaluated by managers. Construct validity not reported. *α* = 0.94–0.96
	Wicaksono et al. (2023)	Individual Innovation Capability Scale (De Jong and Den Hartog 2008)	To measure the nurse's innovation ability. NM	6 items	Content validity not reported. RMSEA = 0.028; TLI = 0.988. Reliability not reported
Organisational innovation culture and implementation	Kim and Park (2015)	Innovative organisational culture scale (Kwon 2011; Kim and Lee 1997).	To measure innovative organisational culture. A 5‐point Likert‐type scale from 1 = strongly disagree to 5 = strongly agree. Higher scores indicate greater levels of innovative organisational culture	5 items	Content validity and construct validity not reported. *α* = 0.88
	Li‐Ying et al. (2016)	Nurse innovation scale, employee version (Welbourne et al. 1998)	To assess the development of novel and useful ideas and the implementation and application of these ideas. A 5‐point Likert scale	4 items, general innovation performance: coming up with new ideas, working to implement new ideas, finding improved ways to do things, creating better processes and routines	Pilot study. Construct validity not reported. *α* = 0.88
	Timmermans et al. (2013)	NRS‐2002 implementation fidelity scale (Polit and Beck 2003)	To determine the implementation effectiveness of the incremental and the radical innovations. A 4‐point Likert Scale from 1 = totally disagree to 4 = totally agree	20 items divided into 2 subscales: Knowledge of the protocol Use of the nutrition protocol in daily practice	Content validity and construct validity not reported. *α* = 0.81–0.85
		Lowry‐Jopp Neuman Model Evaluation Instrument (Lowry 1998; Marrs and Lowry 2006)	To assess the implementation effectiveness of the radical innovation. A 5‐point Likert Scale from 1 = never to 5 = very often	90 items divided into 2 subscales: Knowledge Daily nursing practice	Content validity and construct validity not reported. *α* = 0.91–0.96
Problem‐solving for innovation	Gomes et al. (2015)	Individual Innovation scale (West 1987) translated to Portuguese (Curral and Marques‐Quinteiro 2009)	To indicate the extent to which they had introduced new and improved ways of doing things at work. A 5‐point scale that ranged from 1 = never to 5 = always	5 items (e.g., introduced new methods to meet work targets)	Content validity not reported. RMSEA = 0.073; CFI = 0.994; GFI = 0.990. *α* = 0.87
	Toscano et al. (2023)	Problem‐solving style questionnaire, PSSQ (Cassidy and Burnside 1996)	To measure creative style. A 3‐value forced‐choice format, 0 = False, 0.5 = I am unsure, 1 = True. A higher score indicated a higher level of creative style	4 items	Content validity and construct validity not reported. *α* = 0.83

Abbreviation: NM, not mentioned.

## 3 Results

### 3.1 Characteristics of the Studies

The original studies included in the review were conducted in Belgium (Timmermans et al. 2013), Brazil (Oliveira et al. 2023), China (Lin et al. 2023; Song et al. 2023; Wang et al. 2023), Denmark (Li‐Ying et al. 2016), Egypt (Abdelwahab Ibrahim El‐Sayed et al. 2024), Germany (Binnewies et al. 2007), Indonesia (Wicaksono et al. 2023), Italy (Mura et al. 2016; Toscano et al. 2023), Malaysia (Aoun and Hasnan 2017; Aoun et al. 2018), Portugal (Gomes et al. 2015), South Korea (Kim and Park 2015), Taiwan (Tsai et al. 2013; Weng et al. 2012) and Thailand (Afsar et al. 2018).

The participants of these studies were healthcare professionals (*n* = 6922), managerial nurses (*n* = 30), team leaders (*n* = 121), and research specialist staff (*n* = 15). They worked in various healthcare settings, including diverse hospital environments, such as general hospitals (encompassing public, private, accredited, secondary, lower, tertiary, sharia, university, and affiliated hospitals) operating in different units (surgical, medical, and mixed care), as well as in primary health care, mental health institutions, and hospice and palliative care organisations. Although the review was designed to cover a range of healthcare professions, almost all of the included studies focused on nurses, reflecting the dominance of nursing research in this field.

### 3.2 Healthcare Professionals' Experiences Related to Innovation Competence

The synthesis of qualitative data identified three categories that describe healthcare professionals' experiences of innovation competence (Table 3). These categories were competences for innovation in healthcare, application and influence of innovation in healthcare and challenges and strategies to implementing innovation.

#### 3.2.1 Competences for Innovation in Healthcare

This category describes the key competences that healthcare professionals perceive as essential for healthcare innovation. These competences encompass the development of innovation competence, the ability to integrate innovation into work processes, communication skills, teamwork ability, leadership and management skills, as well as critical thinking and problem‐solving skills.

Developing innovative competences is considered an important part of nurses' postgraduate education. Clinical practice is seen as a key factor in fostering innovative thinking, as participants emphasise that cultivating innovative practical abilities contributes significantly to both their professional growth and the advancement of hospital research (Wang et al. 2023). Integrating innovation into work processes requires adaptation to existing workflows and organisational structures, ensuring that innovative action planning aligns with the practical realities of healthcare settings (Oliveira et al. 2023).

Effective communication skills are regarded as fundamental to the planning and implementation of innovations. Participants highlight that clear and structured communication skills are essential for successfully developing and integrating new practices (Oliveira et al. 2023). Similarly, teamwork abilities are perceived as a vital element in the innovation process, with collaborative efforts and shared knowledge skills playing a key role in service improvements (Oliveira et al. 2023). Leadership and management skills are also considered indispensable, as strong leadership skills provide the strategic vision to guide teams and effectively structure innovation processes (Oliveira et al. 2023). Participants stress that leadership and people management skills form the foundation for innovation, directly influencing nurses' ability to contribute to change within healthcare environments.

Critical thinking and problem‐solving skills were identified as essential for innovation. Participants emphased that the ability to recognise problems and apply analytical thinking is a prerequisite for developing and implementing innovative solutions in nursing practice (Wang et al. 2023).

#### 3.2.2 Application and Impact of Innovation in Healthcare

This category focuses on how innovation is applied in healthcare practice and its effects on healthcare delivery. Innovation is seen as instrumental in enhancing the efficiency and quality of nursing care. Participants emphasised that innovative practical abilities are essential for implementing new projects and maintaining quality control. These skills are particularly valuable in solving clinical problems, ensuring that healthcare interventions remain responsive to evolving patient needs (Wang et al. 2023).

The impact of innovation on healthcare practice is evident in improved methods and processes, with participants noting that innovation enhances overall efficiency. Combining innovative skills with clinical practice is considered crucial, as it allows healthcare professionals to refine their approach and optimise patient care (Wang et al. 2023).

#### 3.2.3 Challenges and Strategies for Implementing Innovation

This category highlights the obstacles encountered in implementing innovation and the strategies used to overcome them. One of the primary challenges identified is the difficulty in translating innovative thinking into practice. Participants note that routine healthcare work often hinders the development and application of innovative ideas, creating a barrier to change (Wang et al. 2023). Addressing these challenges requires structured approaches that facilitate the integration of innovation into daily workflows, ensuring that new ideas can be effectively implemented without disrupting essential healthcare responsibilities (Wang et al. 2023).

### 3.3 Factors Associated With the Innovation Competence of Healthcare Professionals

The systematic review identified a range of factors associated with innovation competence among healthcare professionals (Table 4). These factors were grouped into four categories. These factors were categorised into four groups: sociodemographic characteristics, career‐related characteristics, organisational characteristics and academic factors. These factors were statistically significantly associated (*p* < 0.05 to *p* < 0.001) with a range of outcomes representing innovation competence.

The outcomes related to innovation competence were categorised into three main groups: individual capacities in innovation, innovation‐related competence behaviours, and social and organisational enablers of innovation competence development. The category individual capacities in innovation encompasses the following competences: psychological empowerment (Afsar et al. 2018), information literacy (Lin et al. 2023), individual characteristics (Tsai et al. 2013), and self‐leadership (Gomes et al. 2015). The category innovation‐related competence behaviours includes knowledge sharing (Afsar et al. 2018; Li‐Ying et al. 2016), knowledge‐hiding behaviours (Song et al. 2023) and, work engagement (Gomes et al. 2015). The third category, social and organisational enablers, comprises organisational justice (Song et al. 2023) and nurses' person–organisation fit (Afsar et al. 2018).

Statistically significant sociodemographic characteristics (*p* < 0.05 to *p* < 0.001) included age, gender, relationship status, parental status, and educational background. These factors were associated with various innovation competence outcomes across all three categories namely, individual characteristics, knowledge‐related behaviours and psychological empowerment (Afsar et al. 2018; Gomes et al. 2015; Lin et al. 2023; Li‐Ying et al. 2016; Song et al. 2023; Tsai et al. 2013).

Career‐related characteristics such as years of employment, job tenure, professional title, weekly working hours, and team role were all found to be statistically significantly associated with innovation competence outcomes (*p* < 0.05 to *p* < 0.001). These outcomes included psychological empowerment, information literacy, individual characteristics, self‐leadership, knowledge‐sharing behaviour, knowledge‐hiding behaviour, work engagement, organisational justice, and nurses' person–organisation fit.

In addition, academic engagement factors, including experience in publishing academic papers, participation in training or learning sessions (of at least one day), and time allocated to reading academic literature, also demonstrated significant relationships with specific innovation competence outcomes. These were particularly related to information literacy, knowledge‐hiding behaviour, and organisational justice (Afsar et al. 2018; Gomes et al. 2015; Lin et al. 2023; Song et al. 2023; Tsai et al. 2013; Weng et al. 2012). Organisational characteristics showing statistically significant associations with innovation competence included hospital type (Li‐Ying et al. 2016; Song et al. 2023; Tsai et al. 2013).

Finally, several psychological and behavioural characteristics were also significantly associated (*p* < 0.05 to *p* < 0.001) with innovation competence. These included psychological empowerment, knowledge‐sharing behaviour, self‐leadership, work engagement, information literacy, organisational justice and knowledge‐hiding behaviour (Afsar et al. 2018; Gomes et al. 2015; Lin et al. 2023; Li‐Ying et al. 2016; Song et al. 2023; Tsai et al. 2013; Weng et al. 2012).

### 3.4 Instruments Developed to Assess Innovation Competence and Associated Factors in Healthcare Professionals

The systematic review identified 18 instruments developed to assess innovation competence and associated factors among healthcare professionals. These instruments varied in their focus, structure, and psychometric properties (see Table 5). The Innovative Work Behaviour (IWB) scale (De Jong and Den Hartog 2010) and the Innovative Behaviour Scale for Nurses (Bao et al. 2012) were used in two different studies. In the systematic review, the content validity, construct validity, and reliability of the instruments assessing innovation competence were reported inconsistently (Table 5).

Content validity was reported in 30% of the instruments (Abdelwahab Ibrahim El‐Sayed et al. 2024; Aoun and Hasnan 2017; Aoun et al. 2018; Lin et al. 2023; Song et al. 2023; Tsai et al. 2013), typically assessed through expert panels or content validity indices. Construct validity was also reported in 30% of the instruments (Abdelwahab Ibrahim El‐Sayed et al. 2024; Aoun et al. 2018; Gomes et al. 2015; Toscano et al. 2023; Tsai et al. 2013; Wicaksono et al. 2023), most commonly evaluated using factor analysis and model fit indices such as RMSEA, CFI, and GFI. Reliability was reported in 90% of the instruments (Abdelwahab Ibrahim El‐Sayed et al. 2024; Afsar et al. 2018; Aoun et al. 2018; Binnewies et al. 2007; Gomes et al. 2015; Kim and Park 2015; Lin et al. 2023; Li‐Ying et al. 2016; Mura et al. 2016; Song et al. 2023; Toscano et al. 2023; Tsai et al. 2013; Weng et al. 2012; Timmermans et al. 2013), using internal consistency measures such as Cronbach's *α* or intraclass correlation coefficients. Overall, the quality of instruments reporting can be considered moderate to good. While reliability was addressed with decent statistical methods in most instruments, content and construct validity were less frequently reported.

Instruments can be classified into three broader categories according to the conceptual focus of the outcomes they measure: individual innovation competence, organisational innovation culture and implementation, and problem‐solving for innovation. These instrument categories correspond to the outcome groups identified in Chapter 3.3 of this review.

The category individual innovation competence includes instruments that measure individual‐level innovation outcomes. These instruments assess, for example, innovative behaviour, innovation ability, creativity, creativity of ideas, idea generation, creative self‐efficacy (Abdelwahab Ibrahim El‐Sayed et al. 2024; Afsar et al. 2018; Aoun and Hasnan 2017; Aoun et al. 2018; Binnewies et al. 2007; Kim and Park 2015; Lin et al. 2023; Mura et al. 2016; Song et al. 2023; Toscano et al. 2023; Tsai et al. 2013; Weng et al. 2012; Wicaksono et al. 2023). This category corresponds to the outcome group of individual capacities in innovation described in Chapter 3.3.

The organisational innovation culture and implementation category contains instruments measuring organisation‐level innovation outcomes. These instruments assess innovative organisational culture, the development, implementation and application of novel and valuable ideas, and the effectiveness of implementing both incremental and radical innovations (Kim and Park 2015; Li‐Ying et al. 2016; Timmermans et al. 2013). This category corresponds to the outcome group of social and organisational enablers in innovation competence growth in Chapter 3.3.

The category problem‐solving for innovation includes instruments that assess individuals' cognitive and behavioural approaches to problem‐solving, which contribute to both individual and organisational innovation outcomes. These instruments measure creative problem‐solving style and the extent to which individuals have introduced new and improved ways of doing things at work (Gomes et al. 2015; Toscano et al. 2023). This category corresponds to the outcome group innovation‐related competence behaviours in Chapter 3.3.

### 3.5 Mixed Methodology Outcome on Innovation Competence and Factors Associated With It of Healthcare Professionals

**FIGURE 2:**
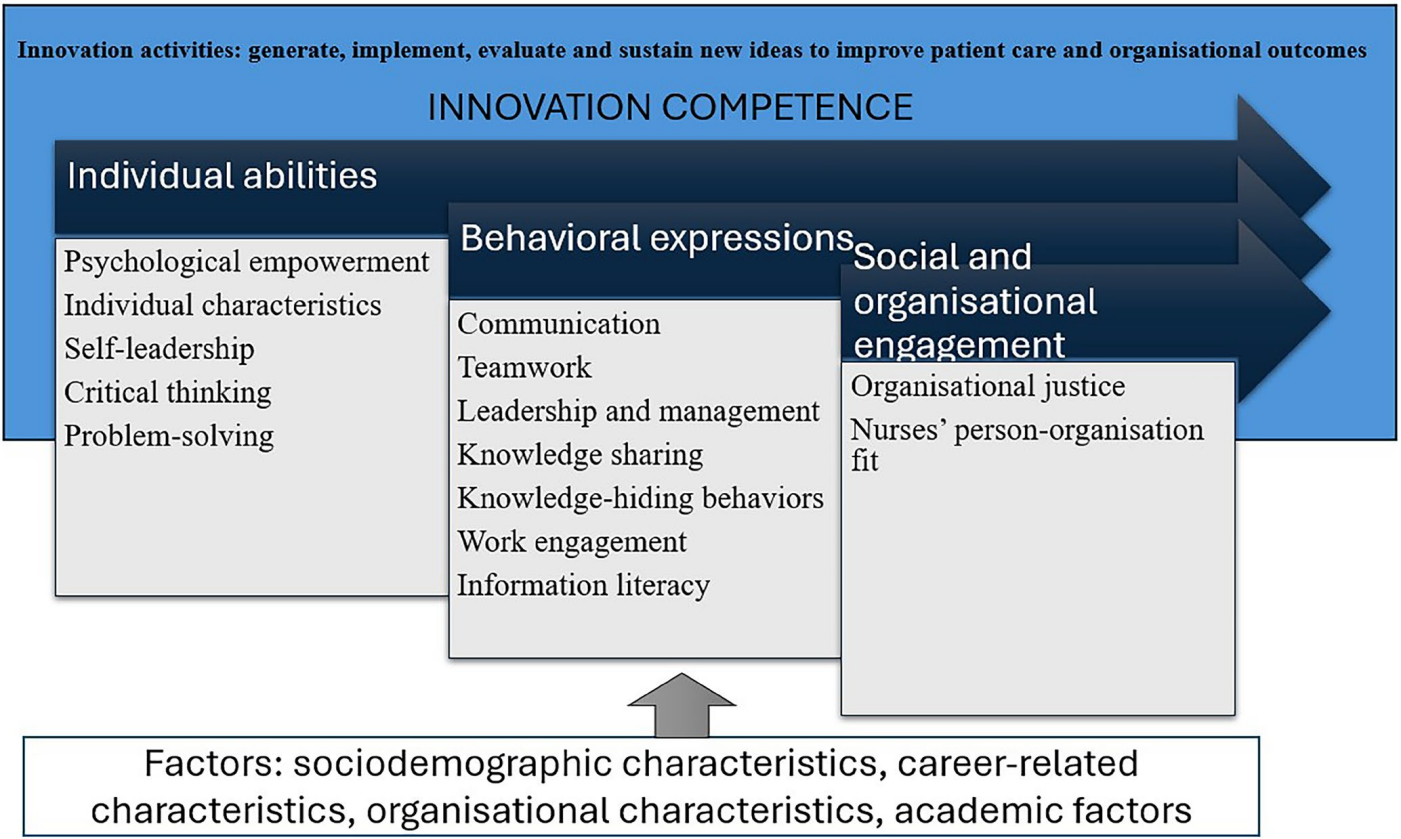
A Multidimensional framework of innovation competence in healthcare professionals. This framework integrates individual abilities, behavioural expressions, and social and organisational engagement as the three areas of innovation competence, based on the synthesis of qualitative and quantitative findings.

As a mixed methodology outcome, the innovation competence among healthcare professionals is defined as a multifaceted encompassing *individual abilities*, *behavioural expressions*, and *social and organisational engagement* that enables the generation, application, and integration of novel ideas and practices to improve healthcare delivery (see Figure 2). Individual abilities are seen by psychological empowerment, individual characteristics, self‐leadership, perceived personal creativity, critical thinking and problem‐solving. Behavioural expressions are presented by communication, teamwork, leadership and management, knowledge sharing, knowledge‐hiding behaviours, work engagement and information literacy. Social and organisational enablers are shown by patient safety climate, organisational justice, perceived worksite support and nurses' person‐organisation fit.

Additionally, empirical findings from both qualitative and quantitative studies highlight that innovation competence is shaped by a range of sociodemographic, career‐related, organisational and academic factors. These include age, gender, relationship status, having children, education, working years, job tenure, professional title, professional experience, hospital experience, papers' reading time, training and learning, number of working hours per week, team role, hospital type, and experience in publishing academic papers. Together, these elements illustrate that innovation competence is not only an individual attribute but also a socially and structurally embedded competence, essential in innovation activities for generating, implementing, evaluating and sustaining new ideas to improve patient care and organisational outcomes.

### 3.6 Ethical Considerations

This mixed‐methods systematic review followed established ethical standards for reviews of previously published literature. The review protocol was registered in the PROSPERO international prospective register of systematic reviews (CRD42024614551) on 10 November 2024. As the study did not involve primary data collection, no further ethical approval was required.

## 4 Discussion

The aim of this systematic review was twofold. First, it sought to identify healthcare professionals' experiences of innovation competence and the factors associated with it. Second, it aimed to examine the instruments that have been developed to assess innovation competence and its associated factors among healthcare professionals. By combining qualitative and quantitative findings, this review offers a multidimensional framework of innovation competence and demonstrates how individual and organisational‐level elements interact in healthcare contexts. A key strength of this review is the move towards constructing a multidimensional, integrated framework of innovation competence in healthcare. This framework synthesises diverse findings and provides a comprehensive perspective through which individual, behavioural, and organisational factors can be understood as interrelated dimensions. As most of the included studies were conducted in nursing, the framework primarily reflects evidence from nursing contexts, even though the review was designed to cover healthcare professionals more broadly.

Innovation activities refer to the generation, implementation, evaluation, and maintenance of new ideas aimed at improving patient care and organisational outcomes (Asurakkody and Shin 2018; Bozkurt and Ercan 2022). By engaging in innovation activities, healthcare professionals contribute to addressing systemic challenges, optimising care processes, and enhancing the quality of patient care (Asurakkody and Shin 2018; Bozkurt and Ercan 2022). Positioned at the frontline of clinical practice, healthcare professionals are uniquely placed to identify practical problems and develop novel solutions (White et al. 2016). Their close interaction with patients also provides a valuable perspective, enabling them to incorporate patient needs directly into innovation efforts (Leary et al. 2022).

In previous literature, innovation competence has been defined as a holistic capability that integrates future‐oriented thinking, creativity, leadership, social collaboration, and practical problem‐solving (Hero et al. 2017; Tidd and Bessant 2009). It encompasses a combination of knowledge, skills, attitudes, values, and behaviours necessary to transform ideas into meaningful solutions. Previous studies have identified adaptability, opportunity recognition, and innovation‐related behaviours as key elements of innovation competence (BDO 2019; Brophy and Kiely 2002; Bozkurt and Ercan 2022; Hero et al. 2017; Pillay and Morris 2016; Tidd and Bessant 2009).

Our review complements previous definitions of innovation competence by offering a new, more systematic perspective on conceptualising the construct. While earlier literature has identified various individual‐level cognitive, interpersonal, and behavioural components of innovation competence, such as creativity, teamwork, and leadership, these elements have often been examined in isolation rather than as part of a comprehensive framework (Bozkurt and Ercan 2022; Hero et al. 2017; Pillay and Morris 2016; Tidd and Bessant 2009; White et al. 2016). Our review contributes by constructing a multidimensional framework that identifies and integrates the interrelated areas of innovation competence and the factors shaping them. In addition, the review highlighted a range of sociodemographic, career‐related, organisational, and academic factors that shape innovation competence. This integrated perspective demonstrates that innovation competence is not only an individual attribute but also embedded in professional roles, workplace interactions, and organisational structures.

The findings of this review highlight several individual abilities that underpin innovation competence, primarily reflecting the nursing perspective as the majority of the included studies focused on nurses. For example, self‐leadership skills have emerged as an important competence area that enables innovation. Healthcare professionals with strong self‐leadership set autonomous goals and actively seek ways to improve care delivery (Gomes et al. 2015; Kim and Park 2015). A positively oriented healthcare professional is psychologically flexible and able to see things from alternative perspectives, which promotes innovative behaviour (Toscano et al. 2023). Problem‐solving skills were also recognised as a component of innovation competence (Oliveira et al. 2023; Wang et al. 2023).

In addition to cognitive and personal abilities, innovation competence is reflected in behavioural expressions that facilitate innovation in practice. As most of the evidence comes from nursing studies, these behavioural competences should be interpreted primarily in the context of nursing practice. Healthcare professionals perceive innovation competence as comprising managerial and leadership skills, communication skills, and teamwork abilities (Oliveira et al. 2023; Wang et al. 2023). Leadership behaviours also play a significant role in innovation competence. Nurse managers have high innovation ability scores because they possess greater leadership and decision‐making authority than staff nurses and have more access to platforms and opportunities to acquire innovation‐related knowledge and resources (Song et al. 2023). Communication competence plays a crucial role in all stages of the innovation process, including preparation, idea generation, and validation, enabling knowledge exchange and collaborative insight (Binnewies et al. 2007). Also, knowledge‐sharing skills were found to be strongly associated with innovative behaviour. Both peer‐to‐peer and hierarchical knowledge exchanges contribute to the ability to generate, develop, and implement innovations (Abdelwahab Ibrahim El‐Sayed et al. 2024; Li‐Ying et al. 2016; Mura et al. 2016; Wicaksono et al. 2023). Information literacy promotes innovative behaviour because it enables the effective integration of information into the knowledge structure, allowing for critical thinking. Reverse thinking, multidirectional thinking, and forward‐looking thinking are the foundations of innovative thinking. Healthcare generates a vast amount of information, so innovation is difficult without information literacy (Lin et al. 2023). Building on these competences, it is essential to recognise that innovation behaviour is a complex, multi‐stage process (Lin et al. 2023), which highlights the evolving nature of both individual capabilities and organisational mechanisms in fostering effective innovation in healthcare.

The findings also emphasise the role of social and organisational engagement as an enabler of innovation competence. Given that the vast majority of included studies were nursing studies, these findings particularly reflect organisational enablers relevant to nursing contexts. Social and organisational enablers, such as organisational justice (Song et al. 2023) and nurses' person–organisation fit (Afsar et al. 2018), underscore the importance of the broader work environment in facilitating innovation competence. According to research by Song et al. (2023), organisational justice supports innovation competence by fostering trust: when nurses perceive fairness in the distribution of work outcomes, they are more likely to trust their organisation, invest in their work, and engage in innovative behaviour by leveraging work autonomy and managing knowledge strategically. Furthermore, perceptions of informational and organisational fairness may empower nurses to elevate their professional voice and status and participate actively in innovation activities (Song et al. 2023). Nurses' person–organisation fit, particularly perceived value congruence, also positively influences how they experience the work environment, fostering a sense of empowerment and encouraging engagement in innovative work behaviour (Afsar et al. 2018). These areas not only complement the more specific competences addressed earlier but also highlight the multifaceted nature of innovation competence, suggesting that both individual attributes and organisational contexts are integral to fostering effective innovation in healthcare settings.

This review also identified multiple sociodemographic, career‐related, organisational, and academic factors associated with innovation competence. Quantitative findings highlighted several sociodemographic and professional factors that are associated with innovation competence, such as age, education, job tenure, clinical experience, and academic activity (Afsar et al. 2018; Gomes et al. 2015; Lin et al. 2023; Song et al. 2023; Tsai et al. 2013). Notably, innovation ability increases with age and experience, underlining the value of accumulated clinical insight. Innovation ability is the capacity to produce new knowledge, ideas, methods, and results by modifying and processing acquired knowledge and experiences through scientific thinking (Lin et al. 2023). Academic engagement, such as publishing scientific papers, was also found to strengthen innovation competence by promoting structured problem‐solving and iterative development (Li‐Ying et al. 2016; Lin et al. 2023).

Despite these enabling factors, several barriers to innovation were identified. These include limited innovation training in clinical practice, routine‐dominated workflows, and knowledge‐hiding behaviours (Song et al. 2023; Wang et al. 2023). Organisational support, clear communication, and team collaboration were noted as critical to overcoming these challenges (Li‐Ying et al. 2016; Oliveira et al. 2023). The compatibility between team learning activities and innovation helps nursing teams improve the effectiveness of implementing various innovations (Timmermans et al. 2013). A positive correlation exists between organisational fairness and innovation ability (Song et al. 2023).

Although multiple instruments have been developed to assess the innovation competence of healthcare professionals, their validity and reliability are inconsistently reported. While some instruments demonstrate high psychometric quality, the lack of standardisation remains a concern (De Jong and Den Hartog 2010; Bao et al. 2012). This finding underscores the need for further development and validation of context‐specific instruments that can accurately capture the multidimensional nature of innovation competence of healthcare professionals. Particularly, as current instruments have been tested mainly in nursing populations, their applicability to other healthcare professions remains to be further examined.

### 4.1 Limitations

This study has several limitations. Our systematic review found that most research on innovation competence in healthcare focuses on nurses, limiting generalisability to other professional groups. Broader studies are needed to better understand innovation competence across the healthcare sector.

The reviewed studies were conducted in various countries, introducing contextual variation in healthcare systems and innovation policies, which may limit the generalisability of findings to specific regions or settings. An interesting finding of this review was that none of the included studies were from English‐speaking countries, despite the extensive search strategy. This suggests that innovation competence in healthcare may still be an under‐researched area in these contexts. By contrast, in several Asian countries such as China, innovation has been a long‐standing policy priority, supported by strategic national programmes and sustained investments in research and development (Li and Zhang 2014; OECD 2008), which may explain why innovation competence has also been more actively studied there. This finding highlights the need for broader international research efforts on innovation competence in healthcare.

Although a mixed‐methods approach was applied, the evidence base was dominated by quantitative studies; only two qualitative studies met the inclusion criteria. This limits the understanding of contextual influences and lived experiences. More qualitative research is needed to explore how innovation is perceived, developed, and supported in practice.

The inclusion criteria were limited to peer‐reviewed studies published in English and Finnish, which may have excluded relevant research published in other languages. Measurement tools varied widely across studies, which may limit the comparability and robustness of findings.

Potential data overlaps may affect the independence of results. For example, Aoun and Hasnan (2017) and Aoun et al. (2018) may have used the same data sources, which could compromise data independence in this review.

## 5 Conclusion

This review underscores the multidimensional nature of innovation competence and the current lack of comprehensive instruments to assess it effectively in healthcare contexts. Future research should focus on developing validated instruments that capture the complexity of innovation competence and on conducting foundational qualitative studies to explore its contextual expressions in practice. In particular, more studies are needed beyond nursing to capture perspectives from other healthcare professions, as well as broader international research to ensure geographically diverse and comparable evidence.

To enhance innovation competence, educational and organisational strategies should adopt a systematic and multilevel approach. These efforts should target not only individual competences, such as leadership, communication, teamwork, knowledge‐sharing, and problem‐solving, but also the broader contextual conditions that influence professional engagement and performance. Embedding innovation competence into professional education and continuing training, as well as into organisational leadership and policy frameworks, could strengthen healthcare systems' ability to sustain innovation capacity.

Strengthening innovation competence also supports the commercialisation of healthcare innovations and may contribute to national health technology development and system sustainability. Investing in innovation competence at both individual and organisational levels can improve the healthcare sector's capacity to address challenges such as population ageing, the ageing of the healthcare workforce, difficulties in attracting new professionals (traction), and in retaining experienced staff (retention), as well as broader economic constraints. Healthcare leaders and policy makers should therefore consider innovation competence as a strategic resource in workforce planning and health system development.

## Author Contributions

All authors have agreed on the final version and meet at least one of the following criteria (recommended by the ICMJE): (1) substantial contributions to conception and design, acquisition of data, or analysis and interpretation of data; (2) drafting the article or revising it critically for important intellectual content. **Mari Saukkoriipi:** conceptualisation, methodology, formal analysis, investigation, writing – original draft, visualisation. **Outi Kanste:** investigation, methodology, conceptualisation, writing – review and editing. **Erika Jarva:** investigation, methodology, conceptualisation, writing – review and editing. **Pauliina Hyrkäs:** investigation, writing – review and editing. **Kristina Mikkonen:** conceptualisation, methodology, formal analysis, investigation, visualisation, writing – review and editing, supervision.

## Funding

The authors have nothing to report.

## Disclosure

Statistical statement: The authors affirm that the methods used in the data analyses are suitably applied to their data within their study design and context, and the statistical findings have been implemented and interpreted correctly.

## Ethics Statement

The authors have nothing to report.

## Conflicts of Interest

The authors declare no conflicts of interest.

## Supporting information


**File S1:** jan70396‐sup‐0001‐FileS1.docx.

**File S2:** jan70396‐sup‐0002‐FileS2.docx.

## Data Availability

The data supporting the findings of this study are available in the published studies included in the review. No new data were generated or analysed in this study.

## References

[jan70396-bib-0001] Abdelwahab Ibrahim El‐Sayed, A. , N. Ahmed Mohamed Elbassal , S. Ahmed Alsenany , S. Mohammed Farghaly Abdelaliem , and M. Alamri . 2024. “Leveraging Supervisor Knowledge Sharing Behavior and Organizational Absorptive Capacity on Nurses' Creativity.” Journal of Nursing Management 2024: 1–13. 10.1155/2024/5480761. PMC11919019 40224821

[jan70396-bib-0002] Afsar, B. , S. Cheema , and B. Bin Saeed . 2018. “Do Nurses Display Innovative Work Behavior When Their Values Match With Hospitals' Values?” European Journal of Innovation Management 21, no. 1: 157–171. 10.1108/EJIM-01-2017-0007.

[jan70396-bib-0003] American Nurses Association . 2017. Innovation. Nursing World. https://www.nursingworld.org/practice‐policy/innovation/events/.

[jan70396-bib-0004] Aoun, M. , and N. Hasnan . 2017. “Health‐Care Technology Management: Developing the Innovation Skills Through Implementing Soft TQM Among Lebanese Hospitals.” Total Quality Management & Business Excellence 28, no. 1–2: 1–11. 10.1080/14783363.2015.1043881.

[jan70396-bib-0005] Aoun, M. , N. Hasnan , and H. Al‐Aaraj . 2018. “Relationship Between Lean Practices, Soft Total Quality Management and Innovation Skills in Lebanese Hospitals.” Eastern Mediterranean Health Journal 24, no. 3: 269–276. 10.26719/2018.24.3.269. 29908022

[jan70396-bib-0006] Aromataris, E. , and Z. Munn , eds. 2020. JBI Manual for Evidence Synthesis. Joanna Briggs Institute. 10.46658/JBIMES-20-01.

[jan70396-bib-0007] Asurakkody, T. A. , and S. Y. Shin . 2018. “Innovative Behavior in Nursing Context: A Concept Analysis.” Asian Nursing Research 12, no. 4: 237–244. 10.1016/j.anr.2018.11.003. 30471386

[jan70396-bib-0008] Bao, L. , L. Wang , and Y. Q. Zhang . 2012. “Development and Reliability and Validity Test of Nurse Innovation Behavior Scale.” Journal of Shanghai Jiao Tong University Medical Edition 32, no. 8: 1079–1082. 10.3969/j.issn.1674-8115.2012.08.025.

[jan70396-bib-0009] Başkurt, E. , and N. A. Ateş . 2020. “Innovation Approaches in Field Area Midwifery.” ERÜ Journal of Health Sciences 7, no. 2: 29–34.

[jan70396-bib-0010] BDO . 2019. “Unleashing Nursing‐Led Innovation.” https://www.bdo.com/insights/industries/healthcare/unleashing‐nurse‐led‐innovation.

[jan70396-bib-0011] Bergendahl, M. , and M. Magnusson . 2015. “Creating Ideas for Innovation: Effects of Organizational Distance on Knowledge Creation Processes.” Creativity and Innovation Management 24, no. 1: 87–101. 10.1111/caim.12097.

[jan70396-bib-0012] Bergevoet, R. M. , and C. Van Woerkum . 2006. “Improving the Entrepreneurial Competencies of Dutch Dairy Farmers Through the Use of Study Groups.” Journal of Agricultural Education and Extension 12, no. 1: 25–39. 10.1080/13892240600723629.

[jan70396-bib-0013] Binnewies, C. , S. Ohly , and S. Sonnentag . 2007. “Taking Personal Initiative and Communicating About Ideas: What Is Important for the Creative Process and for Idea Creativity?” European Journal of Work and Organizational Psychology 16, no. 4: 432–455. 10.1080/13594320701514728.

[jan70396-bib-0014] Birken, S. A. , S. D. Lee , B. J. Weiner , M. H. Chin , and C. T. Schaefer . 2013. “Improving the Effectiveness of Health Care Innovation Implementation: Middle Managers as Change Agents.” Medical Care Research and Review 70, no. 1: 29–45. 10.1177/1077558712457427. 22930312 PMC3727966

[jan70396-bib-0015] Bozic Yams, N. 2017. “Integrated Model of Innovative Competence.” In Proceedings of the 6th International Scientific Conference on Project Management in the Baltic Countries, 21–29. University of Latvia.

[jan70396-bib-0016] Bozkurt, Ö. , and A. Ercan . 2022. “The Effect of Organizational Support on Employees' Innovative Business Behaviors and Intention to Turnover: A Research on the Manufacturing Industry.” İstanbul Commerce University Journal of Social Sciences 21, no. 43: 269–290. 10.46928/iticusbe.1059273.

[jan70396-bib-0017] Brophy, M. , and T. Kiely . 2002. “Competencies: A New Sector.” Journal of European Industrial Training 26, no. 2/3/4: 165–176. 10.1108/03090590210422089.

[jan70396-bib-0018] Bunpin, J. J. , S. Chapman , M. Blegen , and J. Spetz . 2016. “Differences in Innovative Behavior Among Hospital‐Based Registered Nurses.” Journal of Nursing Administration 46, no. 3: 122–127. 10.1097/NNA.0000000000000317. 26866324

[jan70396-bib-0019] Can, E. , B. Dalcal , and H. Durgun . 2023. “Validity and Reliability of Innovative Behaviors of Nurses and Midwives Scale: A Scale Development Study.” Annals of Medical Research 30, no. 9: 1. 10.5455/annalsmedres.2023.01.014.

[jan70396-bib-0020] Carmeli, A. , and J. Schaubroeck . 2007. “The Influence of Leaders and Other Referents' Normative Expectations on Individual Involvement in Creative Work.” Leadership Quarterly 18, no. 1: 35–48. 10.1016/j.leaqua.2006.11.001.

[jan70396-bib-0021] Cassidy, T. , and E. Burnside . 1996. “Cognitive Appraisal, Vulnerability and Coping: An Integrative Analysis of Appraisal and Coping Mechanisms.” Counseling Psychology Quarterly 9, no. 3: 227–243. 10.1080/09515079608256751.

[jan70396-bib-0022] Curral, L. , and P. Marques‐Quinteiro . 2009. “Self‐Leadership and Work Role Innovation: Testing a Mediation Model With Goal Orientation and Intrinsic Motivation.” Revista de Psicología del Trabajo y de Las Organizaciones 25, no. 2: 163–174.

[jan70396-bib-0023] Dai, F. , K. Wei , Y. Chen , and M. Ju . 2019. “Construction of an Index System for Qualitative Evaluation of Undergraduate Nursing Students' Innovative Ability: A Delphi Study.” Journal of Clinical Nursing 28, no. 23–24: 4379–4388. 10.1111/jocn.15020. 31411352

[jan70396-bib-0024] De Jong, J. P. , and D. Den Hartog . 2010. “Measuring Innovative Work Behavior.” Creativity and Innovation Management 19, no. 1: 23–36. 10.1111/j.1467-8691.2010.00547.x.

[jan70396-bib-0025] De Jong, J. P. J. , and D. N. Den Hartog . 2008. “Innovative Work Behavior: Measurement and Validation.” *Scientific Analysis of Entrepreneurship and SMEs*. https://www.eim.nl/smes‐and‐entrepreneurship/research‐reports/.

[jan70396-bib-0027] Gardner, G. , K. Woollett , N. Daly , B. Richardson , and L. M. Aitken . 2010. “Innovation in Clinical Learning for the Acute Hospital Environment: Nursing Grand Rounds.” Nurse Education Today 30, no. 8: 737–741. 10.1016/j.nedt.2010.01.006. 20362365

[jan70396-bib-0028] George, J. M. , and J. Zhou . 2001. “When Openness to Experience and Conscientiousness Are Related to Creative Behavior: An Interactional Approach.” Journal of Applied Psychology 86, no. 3: 513–524. 10.1037/0021-9010.86.3.513. 11419810

[jan70396-bib-0029] Gomes, C. , L. Curral , and A. Caetano . 2015. “The Mediating Effect of Work Engagement on the Relationship Between Self‐Leadership and Individual Innovation.” International Journal of Innovation Management 19, no. 1: 1550009‐1–1550009‐18. 10.1142/S1363919615500097.

[jan70396-bib-0030] Hansen, M. T. , and J. Birkinshaw . 2007. “The Innovation Value Chain.” Harvard Business Review 85, no. 6: 121–130. 17580654

[jan70396-bib-0031] Hero, L. , M. Pitkäjärvi , and K. Matinheikki‐Kokko . 2021. “Validating an Individual Innovation Competence Assessment Tool for University–Industry Collaboration.” Industry and Higher Education 35, no. 4: 485–496. 10.1177/09504222211017447.

[jan70396-bib-0032] Hero, L.‐M. , E. Lindfors , and V. Taatila . 2017. “Individual Innovation Competence: A Systematic Review and Future Research Agenda.” International Journal of Higher Education 6, no. 5: 103–121. 10.5430/ijhe.v6n5p103.

[jan70396-bib-0033] Hyrkäs, P. , L. Haukipuro , S. Väinämö , M. Iivari , A. Sachinopoulou , and J. Majava . 2020. “Collaborative Innovation in Healthcare: A Case Study of Hospitals as Innovation Platforms.” International Journal of Value Chain Management 11, no. 1: 24–41. 10.1504/IJVCM.2020.105475.

[jan70396-bib-0034] Kim, N. H. , and J. H. Lee . 1997. “Empirical Study on Organizational Culture Types, CEO Leadership Styles, and Behavioral Performance.” Korean Journal of Management 5, no. 1: 193–238.

[jan70396-bib-0035] Kim, S. , and M. Park . 2015. “Leadership, Knowledge Sharing, and Creativity: The Key Factors in Nurses' Innovative Behaviors.” Journal of Nursing Administration 45, no. 12: 615–621. 10.1097/NNA.0000000000000274. 26565640

[jan70396-bib-0036] Kleysen, R. F. , and C. T. Street . 2001. “Toward a Multi‐Dimensional Measure of Individual Innovative Behavior.” Journal of Intellectual Capital 2, no. 3: 284–296. 10.1108/EUM0000000005660.

[jan70396-bib-0037] Kwon, J. S. 2011. “The Influence of Innovative Organization Culture on Human Resource Innovation and Organizational Commitment.” Journal of Business Research 23, no. 1: 153–182.

[jan70396-bib-0038] Lahti, N. A. , C. Kevin , S. Schulz , K. Meijers , and G. G. Bothma . 2023. “The Development of the Innovation Readiness Inventory: An Assessment Tool to Assess Innovation Readiness of Nursing Organizations.” SAGE Open Nursing 9: 23779608231202631. 10.1177/23779608231202631. 37745279 PMC10517619

[jan70396-bib-0039] Leary, M. , A. M. Villarruel , and T. S. Richmond . 2022. “Creating an Innovation Infrastructure in Academic Nursing.” Journal of Professional Nursing 38: 83–88. 10.1016/j.profnurs.2021.12.005. 35042594

[jan70396-bib-0040] Li, Y. , and D. Zhang . 2014. Innovation Policy in China (Chapter 3). Economic Research Institute for ASEAN and East Asia. https://www.eria.org/uploads/media/4.ERIA_Innovation_Policy_ASEAN_Chapter_3.pdf.

[jan70396-bib-0041] Lin, M. J. , C. H. Chen , and C. C. Hsu . 2007. “The Supportive Behavior of Supervisor and Coworker on Employee Creativity.” Journal of Technology Management 12, no. 1: 29–63.

[jan70396-bib-0042] Lin, T. , Y. Gao , and X. Feng . 2023. “Relationship Between Information Literacy and Innovative Behavior of Emergency Specialist Nurses: A Cross‐Sectional Study in Southwest China.” International Emergency Nursing 71: 101356. 10.1016/j.ienj.2023.101356. 37972518

[jan70396-bib-0043] Liu, H. Y. , I. T. Wang , N. H. Chen , and C. Y. Chao . 2020. “Effect of Creativity Training on Teaching for Creativity for Nursing Faculty in Taiwan: A Quasi‐Experimental Study.” Nurse Education Today 85: 104293. 10.1016/j.nedt.2019.104293. 31765871

[jan70396-bib-0044] Li‐Ying, J. , M. Paunova , and I. Egerod . 2016. “Knowledge Sharing Behaviour and Intensive Care Nurse Innovation: The Moderating Role of Control of Care Quality.” Journal of Nursing Management 24, no. 7: 943–953. 10.1111/jonm.12404. 27271179

[jan70396-bib-0045] Lizarondo, L. , C. Stern , J. Carrier , et al. 2020. “Chapter 8: Mixed Methods Systematic Reviews.” In JBI Manual for Evidence Synthesis, edited by E. Aromataris and Z. Munn . Joanna Briggs Institute. 10.46658/JBIMES-20-09.

[jan70396-bib-0046] Lockwood, C. , K. Porrit , Z. Munn , et al. 2020. “Chapter 2: Systematic Reviews of Qualitative Evidence.” In JBI Manual for Evidence Synthesis, edited by E. Aromataris and Z. Munn . Joanna Briggs Institute. 10.46658/JBIMES-20-03.

[jan70396-bib-0047] Lowry, L. 1998. The Neuman Systems Model and Nursing Education: Teaching Strategies and Outcomes. Sigma Theta Tau International Centre Nursing Press.

[jan70396-bib-0048] Lucero‐Prisno, D. E. , D. O. Shomuyiwa , M. B. N. Kouwenhoven , et al. 2023. “Top 10 Public Health Challenges to Track in 2023: Shifting Focus Beyond a Global Pandemic.” Public Health Challenges 2, no. 2: e32. 10.1002/pch2.32. PMC12039733 40495865

[jan70396-bib-0049] Marrs, J. A. , and L. W. Lowry . 2006. “Nursing Theory and Practice: Connecting the Dots.” Nursing Science Quarterly 19, no. 1: 44–50. 10.1177/0894318405284124. 16407599

[jan70396-bib-0050] Mikkonen, K. , and M. Kääriäinen . 2020. “Content Analysis in Systematic Reviews.” In The Application of Content Analysis in Nursing Science Research, edited by H. Kyngäs , K. Mikkonen , and M. Kääriäinen , 3–11. Springer. 10.1007/978-3-030-30199-6.

[jan70396-bib-0051] Ministry of Social Affairs and Health . 2022. Sosiaali‐ja Terveydenhuollon Valtakunnalliset Tavoitteet Vuosille 2023–2026 (Sosiaali‐ja Terveysministeriön Julkaisuja 2022:18). Ministry of Social Affairs and Health. https://julkaisut.valtioneuvosto.fi/handle/10024/164547.

[jan70396-bib-0052] Moher, D. , A. Liberati , J. Tetzlaff , and D. G. Altman . 2009. “Reprint—Preferred Reporting Items for Systematic Reviews and Meta‐Analyses: The PRISMA Statement.” Physical Therapy 89, no. 9: 873–880. 10.1093/ptj/89.9.873. 19723669

[jan70396-bib-0053] Moola, S. , Z. Munn , C. Tufanaru , et al. 2020. “Chapter 7: Systematic Reviews of Etiology and Risk.” In JBI Manual for Evidence Synthesis, edited by E. Aromataris and Z. Munn . Joanna Briggs Institute. 10.46658/JBIMES-20-08.

[jan70396-bib-0054] Mura, M. , E. Lettieri , G. Radaelli , and N. Spiller . 2016. “Behavioural Operations in Healthcare: A Knowledge Sharing Perspective.” International Journal of Operations & Production Management 36, no. 10: 1222–1246. 10.1108/IJOPM-04-2015-0234.

[jan70396-bib-0055] National Academies of Sciences, Engineering, and Medicine . 2021. The Future of Nursing 2020–2030: Charting a Path to Achieve Health Equity. National Academies Press. 10.17226/25982. 34524769

[jan70396-bib-0056] OECD . 2008. “OECD Reviews of Innovation Policy: China 2008.” https://www.oecd.org/content/dam/oecd/en/publications/reports/2008/08/oecd‐reviews‐of‐innovation‐policy‐china‐2008_g1gh9531/9789264039827‐en.pdf.

[jan70396-bib-0057] OECD/Eurostat . 2018. Oslo Manual 2018: Guidelines for Collecting, Reporting and Using Data on Innovation. 4th ed. OECD Publishing. 10.1787/9789264304604-en.

[jan70396-bib-0058] Oliveira, I. B. d. , A. M. Peres , R. Almeida Bastos , M. Casey , F. Timmins , and M. Malak . 2023. “Managerial Competencies Engaged in Innovative Actions in Primary Health Care: A Qualitative Study of Brazilian Nurses.” Journal of Nursing Management 2023: 1–9. 10.1155/2023/8746398. PMC11919228 40225651

[jan70396-bib-0059] Pillay, R. , and M. H. Morris . 2016. “Changing Healthcare by Changing the Education of Its Leaders: An Innovation Competence Model.” Journal of Health Administration Education 33, no. 3: 393–410.

[jan70396-bib-0060] Planas‐Campmany, C. , E. Zabaleta‐Del‐Olmo , C. Violán , G. Pérez‐Sánchez , and J. J. Navas‐Palacios . 2020. “Profile of Innovative Ideas Recorded by Nurses in an Ideas Bank of a Corporate Virtual Community of Open Innovation: A Cross‐Sectional Study.” Journal of Nursing Scholarship 52, no. 4: 426–434. 10.1111/jnu.12559. 32346930

[jan70396-bib-0061] Polit, D. F. , and C. T. Beck . 2003. Nursing Research: Principles and Methods. 7th ed. Lippincott Williams & Wilkins.

[jan70396-bib-0062] Popay, J. , H. Roberts , A. Sowden , et al. 2006. Guidance on the Conduct of Narrative Synthesis in Systematic Reviews: A Product From the ESRC Methods Programme. Lancaster University.

[jan70396-bib-0063] Rankin, N. 2004. The New Prescription for Performance: The Eleventh Competency Benchmarking Survey. Competency & Emotional Intelligence Benchmarking, 2004/05.

[jan70396-bib-0064] Rogers, E. M. 1995. Diffusion of Innovations. 4th ed. Free Press.

[jan70396-bib-0065] Scott, S. G. , and R. A. Bruce . 1994. “Determinants of Innovative Behavior: A Path Model of Individual Innovation in the Workplace.” Academy of Management Journal 37: 580–607.

[jan70396-bib-0066] Smith, K. G. , C. J. Collins , and K. D. Clark . 2005. “Existing Knowledge, Knowledge Creation Capability, and the Rate of New Product Introduction in High‐Technology Firms.” Academy of Management Journal 48, no. 2: 346–357. 10.5465/amj.2005.16928421.

[jan70396-bib-0067] Smith, L. Y. 2010. “Creativity Self‐Assessment Questionnaire.” https://lyndismith.files.wordpress.com/2010/05/creativityquestionnaire‐self‐assess.pdf.

[jan70396-bib-0068] Song, J. , Z. Yang , Z. Zhang , and Q. Huang . 2023. “The Correlation Analysis Between Organizational Justice, Knowledge‐Hiding Behaviour and Nurses' Innovation Ability: A Cross‐Sectional Study.” Nursing Open 10, no. 8: 5366–5375. 10.1002/nop2.1774. 37165909 PMC10333867

[jan70396-bib-0069] Tidd, J. , and J. Bessant . 2009. Managing Innovation. 4th ed. Wiley.

[jan70396-bib-0070] Timmermans, O. , R. Van Linge , P. Van Petegem , B. Van Rompaey , and J. Denekens . 2013. “A Contingency Perspective on Team Learning and Innovation in Nursing.” Journal of Advanced Nursing 69, no. 2: 363–373. 10.1111/j.1365-2648.2012.06014.x. 22500919

[jan70396-bib-0071] Toscano, F. , D. Giusino , R. Diana , and T. Rahimi Pordanjani . 2023. “The Role of Emotional Regulation in the Relationship Between Nurses' Creative Style and Innovation Behaviors: A Cross‐Sectional Study.” Nursing Reports (Pavia, Italy) 13, no. 2: 811–822. 10.3390/nursrep13020071. 37368338 PMC10301246

[jan70396-bib-0072] Tsai, H. , S. Liou , Y. Hsiao , and C. Cheng . 2013. “The Relationship of Individual Characteristics, Perceived Worksite Support and Perceived Creativity to Clinical Nurses' Innovative Outcome.” Journal of Clinical Nursing 22, no. 17–18: 2648–2657. 10.1111/jocn.12269. 23710647

[jan70396-bib-0073] Wang, P. , Y. Li , H. Ge , J. Liu , and S. Li . 2023. “Experience in Developing Innovative Practical Ability for Master of Nursing Specialist Degree Program in China: A Qualitative Descriptive Study of Postgraduates.” Nurse Education Today 126: 105811. 10.1016/j.nedt.2023.105811. 37062238

[jan70396-bib-0074] Welbourne, T. M. , D. E. Johnson , and A. Erez . 1998. “The Role‐Based Performance Scale: Validity Analysis of a Theory‐Based Measure.” Academy of Management Journal 41, no. 5: 540–555.

[jan70396-bib-0075] Weng, R. , C. Huang , J. Huang , and M. Wang . 2012. “The Cross‐Level Impact of Patient Safety Climate on Nursing Innovation: A Cross‐Sectional Questionnaire Survey.” Journal of Clinical Nursing 21, no. 15–16: 2262–2274. 10.1111/j.1365-2702.2012.04170.x. 22788560

[jan70396-bib-0076] West, M. A. 1987. “A Measure of Role Innovation in the World of Work.” British Journal of Social Psychology 26, no. 1: 83–85. 10.1111/j.2044-8309.1987.tb00735.x.

[jan70396-bib-0077] White, K. R. , R. Pillay , and X. Huang . 2016. “Nurse Leaders and the Innovation Competence Gap.” Nursing Outlook 64, no. 3: 255–261. 10.1016/j.outlook.2015.12.007. 26827191

[jan70396-bib-0078] Wicaksono, B. S. , A. Hasya , and Sukiman . 2023. “The Impact of Islamic Work Ethics on the Innovative Capability of Nurses: Examining Knowledge Sharing Behavior as an Intermediate Factor (Study at Sultan Agung Islamic Hospital Semarang).” International Research Journal of Innovations in Engineering and Technology 7, no. 11: 716–724. 10.47001/IRJIET/2023.711094.

[jan70396-bib-0079] World Health Organization . 2023. “Health Innovation for Impact.” https://www.who.int/westernpacific/initiatives/innovation‐for‐health‐impact.

[jan70396-bib-0080] Yan, D. , F. Wen , X. Li , and Y. Zhang . 2020. “The Relationship Between Psychological Capital and Innovation Behavior in Chinese Nurses.” Journal of Nursing Management 28, no. 3: 471–479. 10.1111/jonm.12940. 31811781

[jan70396-bib-0081] Yan, J. , J. Yang , Y. Jiang , M. Zhao , and H. Wang . 2018. “Development of Nurse Innovation Ability Scale and Its Reliability and Validity Testing.” Chinese Journal of Nursing 53, no. 10: 1213–1217.

[jan70396-bib-0082] Zhou, J. 1998. “Feedback Valence, Feedback Style, Task Autonomy, and Achievement Orientation: Interactive Effects on Creative Performance.” Journal of Applied Psychology 83, no. 2: 261–276. 10.1037/0021-9010.83.2.261.

[jan70396-bib-0083] Zhou, J. , and G. R. Oldham . 2001. “Enhancing Creative Performance: Effects of Expected Developmental Assessment Strategies and Creative Personality.” Journal of Creative Behavior 35, no. 3: 151–167. 10.1002/j.2162-6057.2001.tb01045.x.

