# Supplementary material for: Innovation Competence in Healthcare: Individual, Environmental and Organisational Factors—A Mixed‐Method Systematic Review
Source: J Adv Nurs. 2026 Jan 16;82(7):7028–57. doi: 10.1111/jan.70396 (PMC13267449; doi:10.1111/jan.70396)
Supplement: Supplementary file 1 — File S1: jan70396‐sup‐0001‐FileS1.docx. [file JAN-82-7028-s001.docx]

Supplementary file 1. Search Terms

| **Data sources, date of search** | Search Terms |
| --- | --- |
| Scopus  1 August 2024 | “Healthcare professional*” OR “health care professional*” OR “healthcare worker*” OR “health care worker*” OR nurse* OR ”nursing staff*” OR midwi* OR physiotherapist* OR “physical therapist*” OR “dental hygienist*” OR “occupational therapist*” OR “healthcare staff” OR “health care staff” OR “healthcare personnel” OR “health care personnel” OR “health professional*” OR “health personnel*” OR ”healthcare worker*” OR ”health care worker*” OR “health worker*” OR “healthcare provider*” OR “health care provider*” OR “health provider*”  AND  (innovation OR innovative* OR creativ*) W/2 (competenc* OR knowledge* OR skill* OR knowhow OR know-how OR expertis* OR abilit* OR aptitude* OR capabilit* OR potential* OR aptitude* OR outcome*) |
| CINAHL  1 August 2024 | Search 1: (MH ”Health Personnel+”) OR “Healthcare professional*” OR “health care professional*” OR “healthcare worker*” OR “health care worker*” OR nurse* OR ”nursing staff*” OR midwi* OR physiotherapist* OR “physical therapist*” OR “dental hygienist*” OR “occupational therapist*” OR “healthcare staff” OR “health care staff” OR “healthcare personnel” OR “health care personnel” OR “health professional*” OR “health personnel*” OR ”healthcare worker*” OR ”health care worker*” OR “health worker*” OR “healthcare provider*” OR “health care provider*” OR “health provider*”  AND  (competenc* OR knowledge* OR skill* OR knowhow OR know-how OR expertis* OR abilit* OR aptitude* OR potential* OR capabilit* OR outcome*) N2 (innovation OR innovative* OR creativ*)  Search 2: (MH ”Health Personnel+”) OR “Healthcare professional*” OR “health care professional*” OR “healthcare worker*” OR “health care worker*” OR nurse* OR ”nursing staff*” OR midwi* OR physiotherapist* OR “physical therapist*” OR “dental hygienist*” OR “occupational therapist*” OR “healthcare staff” OR “health care staff” OR “healthcare personnel” OR “health care personnel” OR “health professional*” OR “health personnel*” OR ”healthcare worker*” OR ”health care worker*” OR “health worker*” OR “healthcare provider*” OR “health care provider*” OR “health provider*”  (MH "Professional Competence+") OR (MH "Professional Knowledge+") OR (MH "Aptitude")  (MH "Diffusion of Innovation") OR (MH "Creativeness") |
| Ovid Medline  1 August 2024 | ”healthcare professional*”[tw] OR ”health care professional*”[tw] OR “health professional*”[tw] OR ”healthcare personnel*”[tw] OR ”health care personnel*”[tw] OR “health personnel*”[tw] OR “Health Personnel”[Mesh] OR ”healthcare worker*”[tw] OR ”health care worker*”[tw] OR “health worker*”[tw] OR “healthcare provider*”[tw] OR “health care provider*”[tw] OR “health provider*”[tw] OR nurse*[tw] OR ”nursing staff*”[tw] OR physician*[tw] OR doctor*[tw] OR clinician*[tw] OR ”medical staff*”[tw] |
| Web of Science  1 August 2024 | “Healthcare professional*” OR “health care professional*” OR “healthcare worker*” OR “health care worker*” OR nurse* OR ”nursing staff*” OR midwi* OR physiotherapist* OR “physical therapist*” OR “dental hygienist*” OR “occupational therapist*” OR “healthcare staff” OR “health care staff” OR “healthcare personnel” OR “health care personnel” OR “health professional*” OR “health personnel*” OR ”healthcare worker*” OR ”health care worker*” OR “health worker*” OR “healthcare provider*” OR “health care provider*” OR “health provider*”  AND  (innovation* OR innovative* OR creativ*) NEAR/2 (competenc* OR knowledge* OR skill* OR knowhow OR know-how OR expertis* OR abilit* OR aptitude* OR capabilit* OR potential* OR aptitude* OR outcome*) |
| ProQuest  1 August 2024 | “Healthcare professional*” OR “health care professional*” OR “healthcare worker*” OR “health care worker*” OR nurse* OR ”nursing staff*” OR midwi* OR physiotherapist* OR “physical therapist*” OR “dental hygienist*” OR “occupational therapist*” OR “healthcare staff” OR “health care staff” OR “healthcare personnel” OR “health care personnel” OR “health professional*” OR “health personnel*” OR ”healthcare worker*” OR ”health care worker*” OR “health worker*” OR “healthcare provider*” OR “health care provider*” OR “health provider*”  AND  (innovation* OR innovative* OR creativ*) NEAR/2 (competenc* OR knowledge* OR skill* OR knowhow OR know-how OR expertis* OR abilit* OR aptitude* OR capabilit* OR potential* OR aptitude* OR outcome*) |
| PsycArticles  1 August 2024 | Search 1: DE "Health Personnel" OR DE "Allied Health Personnel" OR DE "Caregivers" OR DE "Medical Personnel" OR DE "Mental Health Personnel" OR “Healthcare professional*” OR “health care professional*” OR “healthcare worker*” OR “health care worker*” OR nurse* OR ”nursing staff*” OR midwi* OR physiotherapist* OR “physical therapist*” OR “dental hygienist*” OR “occupational therapist*” OR “healthcare staff” OR “health care staff” OR “healthcare personnel” OR “health care personnel” OR “health professional*” OR “health personnel*” OR ”healthcare worker*” OR ”health care worker*” OR “health worker*” OR “healthcare provider*” OR “health care provider*” OR “health provider*”  AND  (innovation* OR innovative* OR creativ*) N2 (competenc* OR knowledge* OR skill* OR knowhow OR know-how OR expertis* OR abilit* OR aptitude* OR capabilit* OR potential* OR aptitude* OR outcome*)  Search 2: (DE "Competence" OR DE "Professional Competence")  (DE "Innovation") OR (DE "Creativity")  #1 AND #3 AND #4  Search 3: Search 1 OR Search 2 |
| Medic  1 August 2024 | “terveydenhuollon henkilökunta” OR sairaanhoi* OR kätil* OR fysioterap* OR toimintaterap* OR suuhyg* OR terveydenhoit* OR “Healthcare professional*” OR “health care professional*” OR “healthcare worker*” OR “health care worker*” OR nurse* OR ”nursing staff*” OR midwi* OR physiotherapist* OR “physical therapist*” OR “dental hygienist*” OR “occupational therapist*” OR “healthcare staff” OR “health care staff” OR “healthcare personnel” OR “health care personnel” OR “health professional*” OR “health personnel*” OR ”healthcare worker*” OR ”health care worker*” OR “health worker*” OR “healthcare provider*” OR “health care provider*” OR “health provider*”  AND  Innov* OR luovuu* OR creativ*  AND  Taito* OR taido* OR kompetens* OR osaam* OR kompetent* OR pystyv* OR kyke* OR kyky OR kyvy* OR pätev* OR competenc* OR knowledge* OR skill* OR knowhow OR know-how OR expertis* OR abilit* OR aptitude* OR capabilit* OR potential* OR aptitude* OR outcome* |
