# Supplementary material for: Innovation Competence in Healthcare: Individual, Environmental and Organisational Factors—A Mixed‐Method Systematic Review
Source: J Adv Nurs. 2026 Jan 16;82(7):7028–57. doi: 10.1111/jan.70396 (PMC13267449; doi:10.1111/jan.70396)
Supplement: Supplementary file 2 — File S2: jan70396‐sup‐0002‐FileS2.docx. [file JAN-82-7028-s002.docx]

Supplementary file 2. Quality Assessment

| The Critical Appraisal Checklist for Analytical Cross-sectional Studies | | | | | | | | | | | | | | | | | | |  |
| --- | --- | --- | --- | --- | --- | --- | --- | --- | --- | --- | --- | --- | --- | --- | --- | --- | --- | --- | --- |
| Study | | Were the criteria for inclusion in the sample clearly defined? | | Were the study subjects and the setting described in detail? | | Was the exposure measured in a valid and reliable way? | | Were objective, standard criteria used for measurement of the condition? | | | Were confounding factors identified? | | Were strategies to deal with confounding factors stated? | | Were the outcomes measured in a valid and reliable way? | | Was appropriate statistical analysis used? | | Total scores, % |
| Abdelwahab Ibrahim El-Sayed et al., 2024 | | YES | | YES | | YES | | YES | | | YES | | YES | | YES | | YES | | 8/8, 100 % |
| Afsar, 2018 | | YES | | YES | | YES | | YES | | | YES | | YES | | YES | | YES | | 8/8, 100 % |
| Aoun & Hasnan, 2017 | | YES | | YES | | UNCLEAR | | NO | | | NO | | NO | | YES | | YES | | 4/8, 50 % |
| Aoun, Hasnan & Al Aaraj, 2018 | | YES | | YES | | YES | | YES | | | YES | | YES | | YES | | YES | | 8/8, 100 % |
| Binnewies, Ohly & Sonnentag, 2007 | | NO | | UNCLEAR | | YES | | YES | | | YES | | YES | | YES | | YES | | 6/8, 75 % |
| Gomes, Curral & Caetano, 2015 | | NO | | YES | | YES | | YES | | | YES | | YES | | YES | | YES | | 7/8, 87,5 % |
| Kim & Park, 2015 | | YES | | YES | | YES | | YES | | | YES | | YES | | YES | | YES | | 8/8, 100 % |
| Lin, Gao & Fen, 2023 | | YES | | YES | | YES | | YES | | | YES | | YES | | YES | | YES | | 8/8, 100 % |
| Li-Ying, Paunova & Egerod, 2016 | | YES | | YES | | YES | | YES | | | YES | | YES | | YES | | YES | | 8/8, 100 % |
| Mura et al., 2016 | | UNCLEAR | | YES | | YES | | YES | | | YES | | YES | | YES | | YES | | 7/8, 87,5 % |
| Song et al., 2023 | | YES | | YES | | YES | | YES | | | YES | | YES | | YES | | YES | | 8/8, 100 % |
| Timmermans et al., 2013 | | YES | | YES | | YES | | YES | | | NO | | NO | | YES | | YES | | 6/8, 75 % |
| Toscano et al., 2023 | | YES | | YES | | YES | | YES | | | YES | | YES | | YES | | YES | | 8/8, 100 % |
| Tsai et al., 2013 | | YES | | YES | | YES | | YES | | | YES | | YES | | YES | | YES | | 8/8, 100 % |
| Weng et al., 2012 | | YES | | YES | | YES | | YES | | | YES | | YES | | YES | | YES | | 8/8, 100 % |
| Wicaksono, Hasya & Sukiman, 2023 | | NO | | YES | | YES | | YES | | | NO | | NO | | YES | | YES | | 5/8, 62,5 % |
| The Critical Appraisal Checklist for Qualitative Studies | | | | | | | | | | | | | | | | | | | |
| Study | Is there congruity between the stated philosophical perspective and the research methodology? | | Is there congruity between the research methodology and the research question or objectives? | | Is there congruity between the research methodology and the methods used to collect data? | | Is there congruity between the research methodology and the representation and analysis of data? | | Is there congruity between the research methodology and the interpretation of results? | Is there a statement locating the researcher culturally or theoretically? | | Is the influence of the researcher on the research, and vice-versa, addressed? | | Are participants, and their voices, adequately represented? | | Is the research ethical according to current criteria, and is there evidence of ethical approval by an appropriate body? | | Do the conclusions drawn in the research report flow from the analysis, or interpretation, of the data? |  |
| Oliveira, 2023 | NO | | YES | | YES | | YES | | YES | NO | | NO | | YES | | YES | | YES | 7/10, 70 % |
| Wang et al., 2023 | YES | | YES | | YES | | YES | | YES | YES | | YES | | YES | | YES | | YES | 10/10, 100 % |
